# Ononin delays the development of osteoarthritis by down-regulating MAPK and NF-κB pathways in rat models

**DOI:** 10.1371/journal.pone.0310293

**Published:** 2024-10-31

**Authors:** Fang Xu, Zhaocong Li, Yueming Jiang, Ting Liao, Michael Aschner, Qingjun Wei

**Affiliations:** 1 Department of Orthopaedics Trauma and Hand Surgery, The First Affiliated Hospital of Guangxi Medical University, Nanning, China; 2 Guangxi Colleges and Universities Key Laboratory of Prevention and Control of Highly Prevalent Diseases, Guangxi Medical University, Nanning, China; 3 Institute of Brain and Mental Diseases, Guangxi Academy of Medical Sciences, The People’s Hospital of Guangxi Zhuang Autonomous Region, Nanning, China; 4 Department of Toxicology, School of Public Health, Guangxi Medical University, Nanning, Guangxi, China; 5 Department of Molecular Pharmacology, Albert Einstein College of Medicine, Bronx, New York, United States of America; 6 Department of Orthopedics, The Second Affiliated Hospital of Guangxi Medical University, Nanning, Guangxi, China; Foshan University, CHINA

## Abstract

**Background:**

Osteoarthritis (OA) is featured as cartilage loss, joint pain and loss of labor, which the inflammatory reaction may play critical roles. Ononin is an isoflavone isolating from medicinal plants and has anti-inflammatory effects. Our study investigated the anti-inflammation response of ononin on OA.

**Methods:**

Anterior cruciate ligament transection (ACLT)–induced OA operation was used to establish research model, then treated with ononin for 8 weeks. The condition of joint injury was assessed using pathological staining. The concentration of tumor necrosis factor-α (TNF-α), interleukin-1β (IL-1β) and interleukin-6 (IL-6) in serum were measured by Elisa kit. The expression of collagen II and matrix metalloproteinase 13 (MMP-13) proteins to assess cartilage metabolism level by immunohistochemistry and Western blot. We detected the expression of proteins involved in the MAPK and NF-κB signaling pathways. Finally, we used molecular docking to assess the affinity of ononin for the target proteins ERK1/2, JNK1/2, p38 and p65.

**Results:**

Our results confirmed that ononin ameliorated cartilage impairment through histopathological analysis by improving the morphological structures and cartilage tidal lines and decreasing Osteoarthritis Research Society International (OARSI) scores in OA rats. Moreover, ononin inhibited the secretion of above factors in OA rats. Furthermore, ononin has been shown to improve cartilage content levels in OA rats. In addition, ononin inhibited the reactivity of MAPK and NF-κB pathways in OA rats. And molecular docking indicated the ligand molecules could stably bind to the proteins of above receptors.

**Conclusion:**

Our results demonstrated that ononin may ameliorate cartilage damage and inflammatory response in OA rats by downgrading MAPK and NF-κB pathways, thus identifying ononin as a potential novel drug to treat OA.

## Introduction

Osteoarthritis (OA) is an uneven, progressive disease featured as articular cartilage injury, cartilage loss, joint pain and physical disability [1]. With the increase in the aging people, the number of arthritis patients has increased, making it a major disability factor in the elderly population and representing the most common joint disease [2, 3]. Current OA treatments, both non-pharmacological and pharmacological, primarily aim at relieving symptoms and improving function but fail to reverse disease progression [4]. There are no satisfactory treatment options for OA except for final joint replacement. Consequently, OA remains a challenge and a major focus of clinical and scientific research [5].

A typical feature of OA is the inflammatory response, evidenced by the detection of several inflammatory factors in patients’ serum, cartilage and synovial fluid [6, 7]. Indeed, inflammation and its cascading reaction mechanisms can accelerate cartilage degeneration and alter the function of cartilage cells [8]. For example, Interleukin-1 β (IL-1β) can induce extracellular matrix (ECM) degradation and activate matrix metalloproteinase in chondrocytes, thereby contributing to cartilage degradation and the progression of OA [9]. MMP13 has been demonstrated to play a pivotal role in degrading ECM components. High expression of MMP-13 can lead to the degradation of collagen II in cartilage [10, 11]. Moreover, the data have been validated, indicating a close association between the MAPK and NF-κB and the pathological process of OA [12], which activated above pathways to release various inflammatory factors [13–15]. Hence, employing this strategy may represent a significant breakthrough in the treatment of OA.

**Fig 1:**
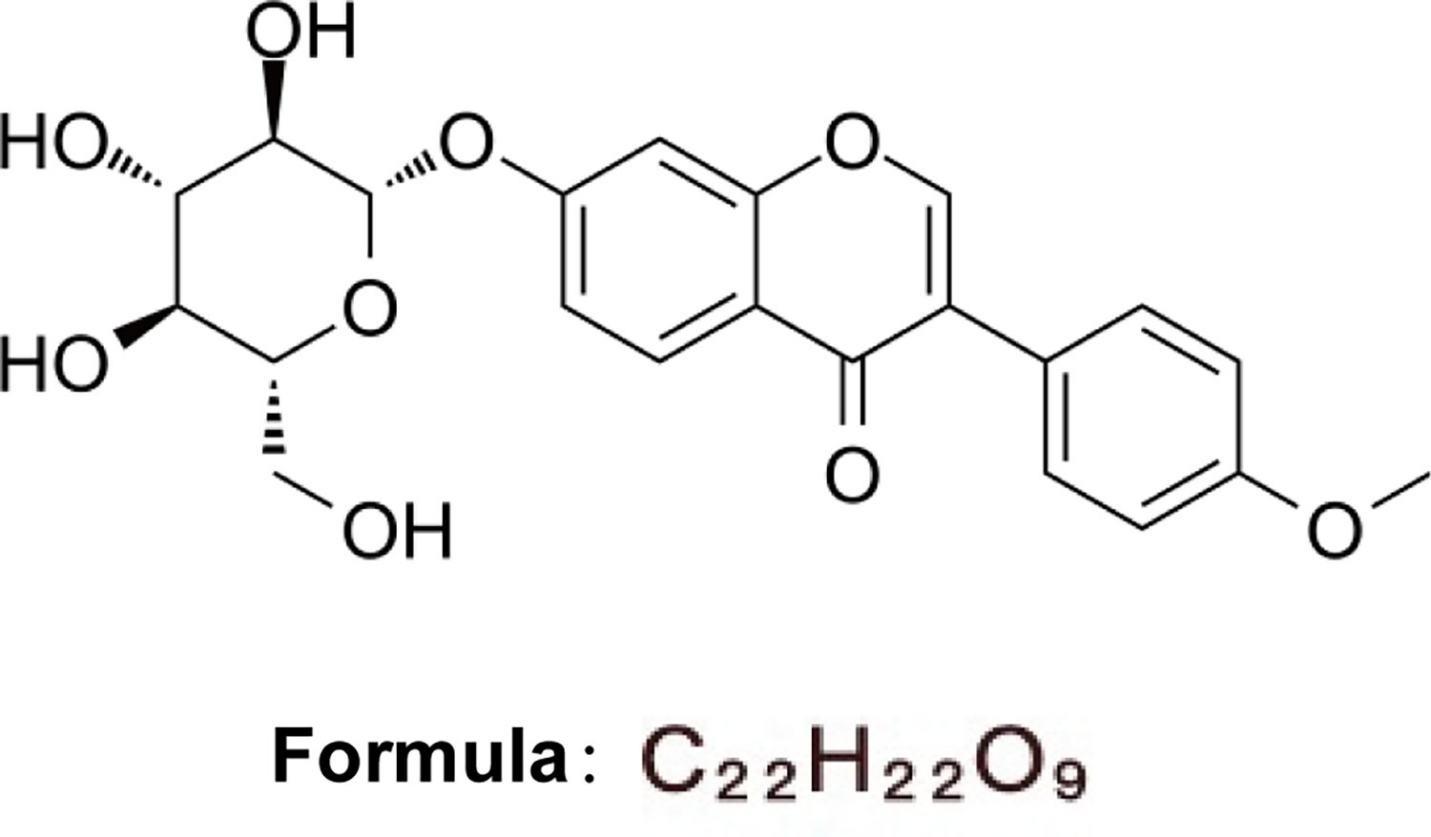
Ononin chemical structure.

Presently, the therapeutic regimen for knee osteoarthritis typically recommends nonsteroidal anti-inflammatory drugs (NSAIDs) for the relief of pain symptoms [4]. However, it is noteworthy that their usage may entail serious side effects on gastrointestinal and cardiovascular health [16, 17]. Consequently, Chinese herbal compounds have demonstrated notable tolerability and safe in knee osteoarthritis patients, presenting a promising complementary option for mitigating the progression of OA [18]. Ononin, (Fig 1) an isoflavone glycoside derived from legumes such as kudzu, lupine, broccoli, cauliflower, and soy, exhibits anti-inflammatory, anti-oxidant, and anti-tumour properties across a spectrum of diseases [19–22]. Chen et al. [23] have reported that ononin improves symptoms of Alzheimer’s disease by mitigating cognitive decline. Furthermore, ononin has been observed to attenuate levels of inflammatory factors by modulating the activities of the NF-κB and MAPK pathways [24]. However, the potential of ononin to ameliorate inflammation in OA remains to be fully elucidated. Thus, our objective is to delineate its underlying mechanism in the context of OA.

## Materials and methods

### Reagents

Dimethylsulfoxide (DMSO) was purchased from Sigma-Aldrich (Missouri, USA). Ononin was bought from MedChemExpress (MCE) (New Jersey, USA). The primary antibodies of p-ERK/ERK, p-p38/p38, p-JNK/JNK, p-p65/p65, p-IκBα/IκBα, secondary antibodies, and protease and phosphatase inhibitors were all provided by Cell Signalling Technology (Beverly, Massachusetts, USA). MMP-13 and Collagen II, and the internal reference antibody GAPDH were purchased from Abcam (Cambridge, UK). Primary and secondary antibody dilutions were purchased from Boster (Wuhan, China). EDTA decalcifying solution, sterile saline, pentasorbital sodium and paraformaldehyde were purchased from Solarbio (Beijing, CN).

### Animals

Fifty male Sprague–Dawley rats (8 weeks old, 160~200 g) were procured from the Laboratory Animal Center of Guangxi Medical University. Our study adhered to international standards of animal care guidelines and received ethical approval (approval number: 202001004). The rats were housed in a specific pathogen-free (SPF)-grade facility at a constant temperature of 24°C and provided with ad libitum access water and food.

### Model preparation and experimental design

**Fig 2:**
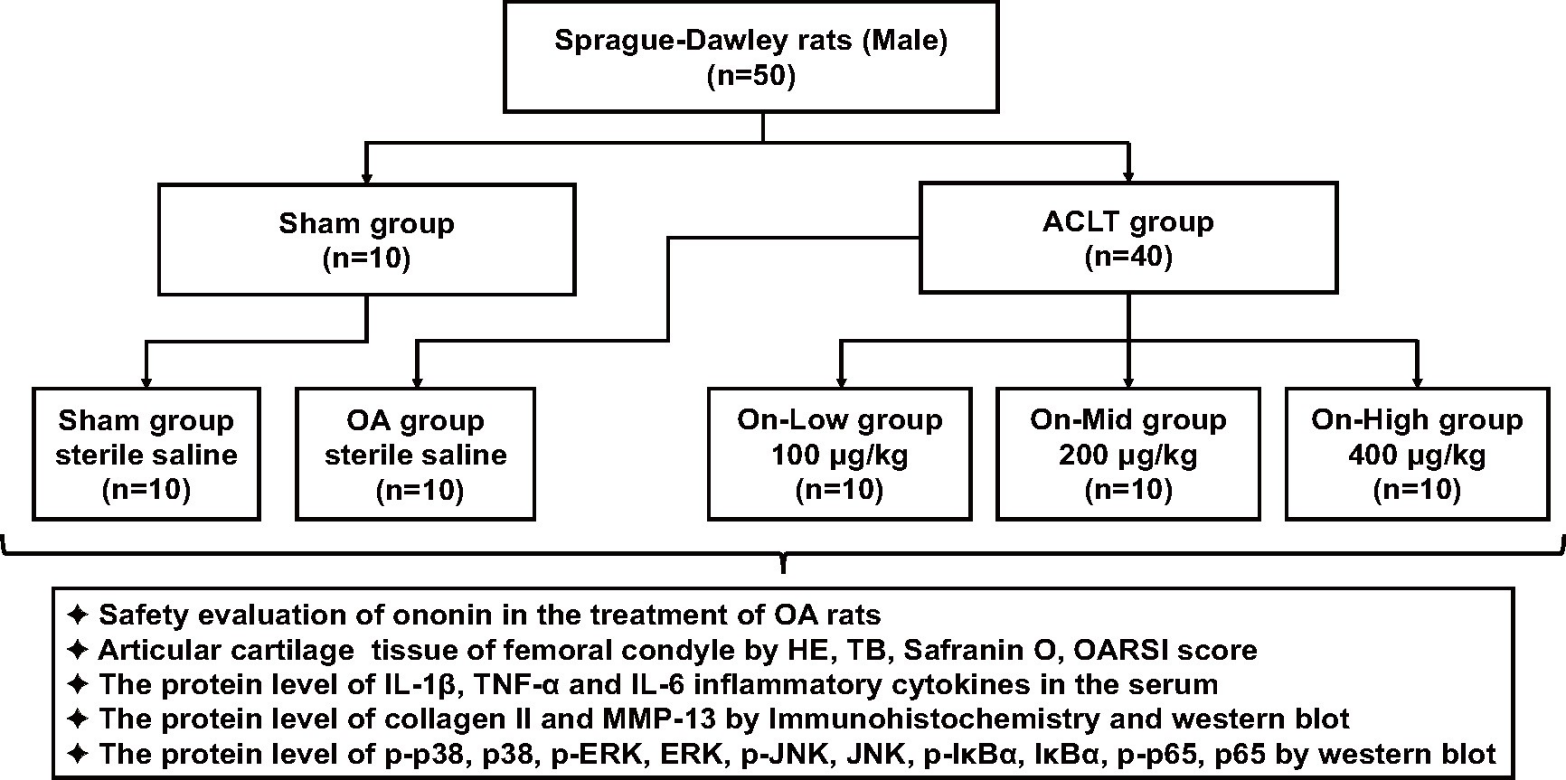
The diagram of experimental design and grouping. Abbreviation: hematoxylin-eosin (HE), toluidine blue (TB), Safranin O (SF), interleukin-1β (IL-1β), tumor necrosis factor-α (TNF-α), interleukin-6 (IL-6), matrix metalloproteinase 13 (MMP-13).

The anterior cruciate ligament transection (ACLT)-induced OA model was prepared using the modified Hulth’s method [25], with anaesthesia administered via intraperitoneal injection of sodium pentobarbital (30 mg/kg). All rats were randomly divided into five groups using a blinding method: Sham group, OA group, On-Low group (100 μg/kg ononin-treated), On-Mid group (200 μg/kg ononin-treated) and On-High group (400 μg/kg ononin-treated). The OA group and ononin-treated groups underwent ACLT surgery. Subsequently, the ononin-treated groups received intra-articular (i.a.) injections of 100, 200, 400 μg/kg ononin once a week for 8 weeks. Throughout the treatment period, the non-treatment group received intra-articularly injections of an equal volume of non-ononin-containing solution. At the conclusion of the study, all rats were euthanized by blood collection under anaesthesia induced by intraperitoneal injection of sodium pentobarbital (30 mg/kg). Serum was obtained by centrifugation of the blood samples and stored at—20°C. Knee joints and samples from the liver, spleen, and kidney were dissected and frozen at—80°C after fixation with 40% formalin. The flowchart outliningthe entire experimental procedure is depicted in Fig 2.

### Safety evaluation of ononin

Body weight and organ coefficient were recorded and measured for each group to assess the toxicity of ononin in rats. Briefly, rat body weights in each group were measured weekly until the end of the treatment period. Organs, including the liver, spleen, and kidney, stored at—80°C, were weighed, and the organ coefficient was calculated using the formula: organ coefficient = (tissue weight/body weight) × 100%. Subsequently, liver, spleen and kidney samples were prepared as paraffin sections and cut into 0.5 cm × 0.5 cm blocks. These samples were stained with hematoxylin and eosin (HE). Two independent and experienced observers assessed the difference of the organ samples under phase-contrast microscopy (Evos Flauto, Life Technologies, USA).

### Superficial observation and pathological examination of cartilage

All knee images were captured using an SLR camera (Canon 750d, Japan) to assess cartilage injury. The major content for superficial observation into the following content: the cartilage surface, the cartilage surface and cartilage erosion. Two independent and experienced observers performed the scoring of the cartilage pathological changes.

Articular cartilage samples from each group were sectioned into 5-μm thicknesses after preparing as paraffin sections to stain toluidine blue (TB), Safranin O (SF) and hematoxylin-eosin (HE). After staining, we evaluated the damage degree of femoral condyle cartilage by the OARSI scoring. Two independent and experienced observers conducted the scoring of cartilage pathological changes.

### Western blot analysis

The cartilage tissue was lysed to extract protein, the levels of which were quantified using the bicinchoninic acid (BCA) kit. Subsequently, after applying target antibodies, the immune response band was detected using the Western ECL system (Boster, CN) with ECL working solution. The density of proteins was analyzed using Image J software, with GAPDH serving as the control.

### ELISA assay

The previously centrifuged serum from each group was thawed to room temperature, and the concentration of inflammatory cytokines was assessed enzyme-linked immunosorbent assay (ELISA). The ELISA procedure was conducted following the instruction provided by R&D Systems company.

### Immunohistochemistry

Immunohistochemical staining was conducted to detect collagen II and MMP-13 proteins in cartilage tissue. Histologic sections were embedded in paraffin, sliced, dewaxed with xylene, and dehydrated using gradient ethanol. The primary antibody working solution, appropriately diluted with 1% BSA antibody diluent, was incubated at 4°C for 12 hours. Following incubation with the secondary antibody, images were obtained acquired a 200x microscope.

### Docking analysis

We employed molecular docking to evaluate the affinity of ononin for the target proteins ERK1/2, JNK1/2, P38, and P65. Initially, we retrieved the structural formula of the ligand molecule from the PubChem database (https://pubchem.ncbi.nlm.nih.gov/) and minimized its molecular energy using Chem3D software. The resulting minimized structure was exported to mol2 format. Subsequently, we downloaded the PDB format structure files of the receptor proteins ERK1 (PDB ID: 2ZOQ), ERK2 (PDB ID: 3C9W), JNK1 (PDB ID: 4L7F), JNK2 (PDB ID: 7CML), and P38 (PDB ID: 4MYG) from the PDB database (https://www1.rcsb.org/). The receptor proteins were preprocessed using PyMOL software, and both the small molecule ligands (in mol2 format) and the receptor proteins (in pdb format) were converted to pdbqt format using AutoDockTools 1.5.6 software. Active pockets within the receptor proteins were identified, and the search conformation range was set accordingly. Finally, the Vina script was executed to calculate the molecular binding energy, and the results of molecular docking were obtained. A Vina binding energy ≤ -5.0 kcal·mol-1 indicates stable docking, while a Vina binding energy ≤ -7.0 kcal·mol-1 indicates very strong binding between the ligand molecule and the receptor protein. The ligand-receptor complexes generated by molecular docking were visualized in 3D using PyMOL software and 2D using Discovery Studio software to assess the reliability of bioinformatics analysis and prediction.

### Statistical analysis

The results in each group were expressed as the mean ± standard deviation (SD). Statistical analysis was performed using GraphPad Prism 10 software, employing One-way analysis of variance (ANOVA) for multiple groups’ data, followed by two-group comparisons using the Tukey Test. P<0.05 denoted statistical significance. Significance levels were denoted as follows: *Indicates P<0.05, **indicates P< 0.01, ***indicates P < 0.001.

## Results

### Safety of ononin in the treatment of OA rats

**Fig 3:**
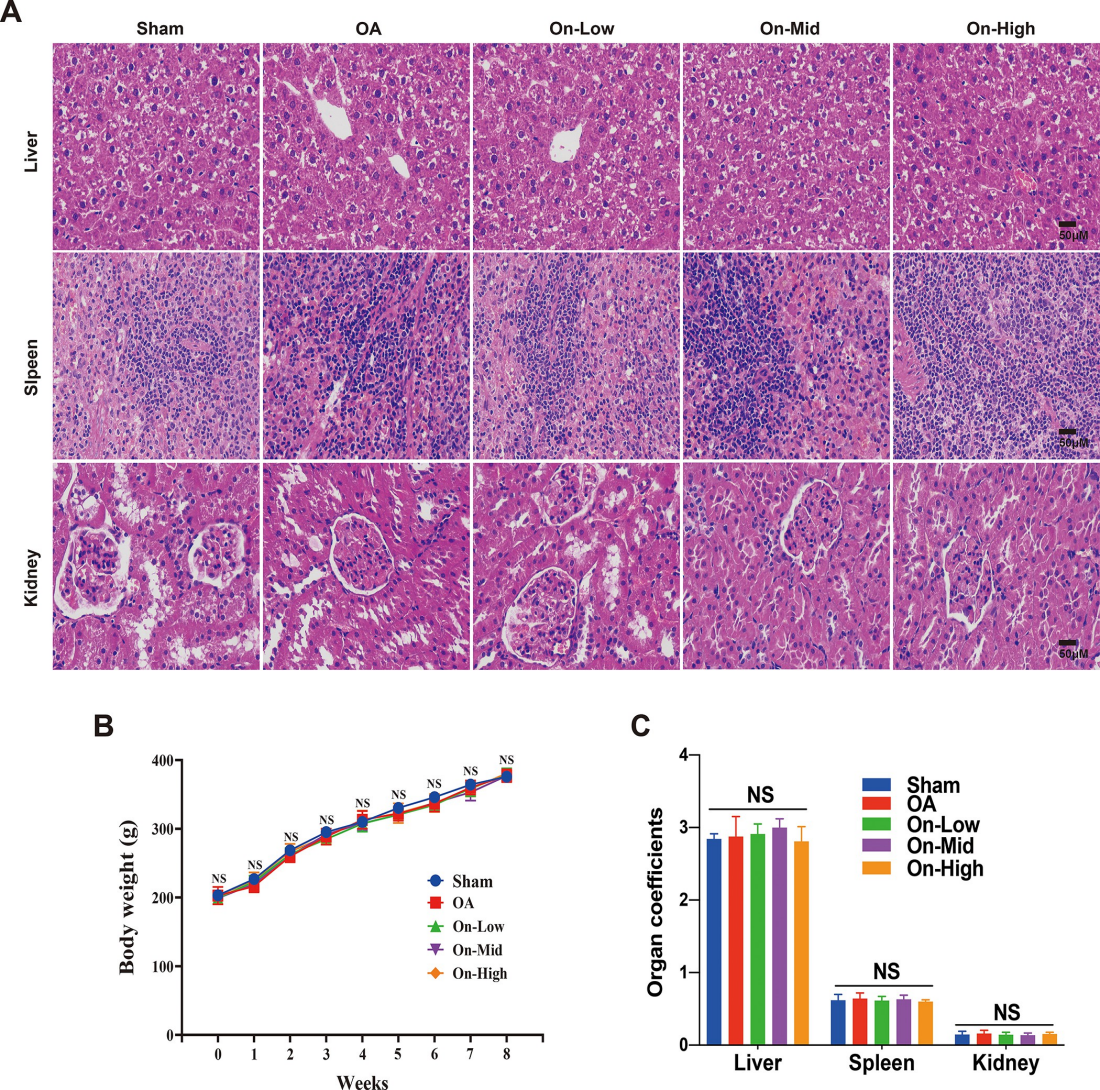
Safety evaluation of ononin in the treatment of OA rats. (A) HE staining of liver, spleen and kidney sections (scale bar: 50 μm). (B) Curve of body weight of rats over time, n = 10 (C) The organ coefficients of liver, spleen and kidney were measured after 8 week of post-surgery, n = 3. **NS**: No significant differences from each group.

To explore whether ononin exhibits any toxic effects, we monitored the body weights of the rats (Table 1 in S2 Raw data), calculated the organ coefficients (Table 2 in S2 Raw data) of spleen, liver, and kidney, and conducted histopathological examinations using HE staining. The histopathological results revealed uniform staining of liver tissues in all groups, with clear structure of liver lobules, neatly arranged liver cords, and intact morphology of liver cells. Similarly, spleen tissue analysis indicated consistent proportions of red pulp and white pulp across all groups, with no apparent abnormalities in tissue morphology. Renal pathology examination demonstrated a clear distribution of renal cortex and medulla, with normal glomerular volume and cell count in the cortex, and no signs of necrosis, vitrification, or fibrosis (Fig 3A). Moreover, as depicted in Fig 3B, the body weights of the rats increased gradually in every group, with no significant differences observed (P>0.05, Table 1 in S1 Raw data). Furthermore, the organ coefficients in all groups yielded similar results (P>0.05, see Fig 3C). In summary, each ononin-treated group exhibited no apparent toxicity in the liver, spleen, or kidney (Table 2 in S1 Raw data).

### Ononin alleviated cartilage damage in OA rats

**Fig 4:**
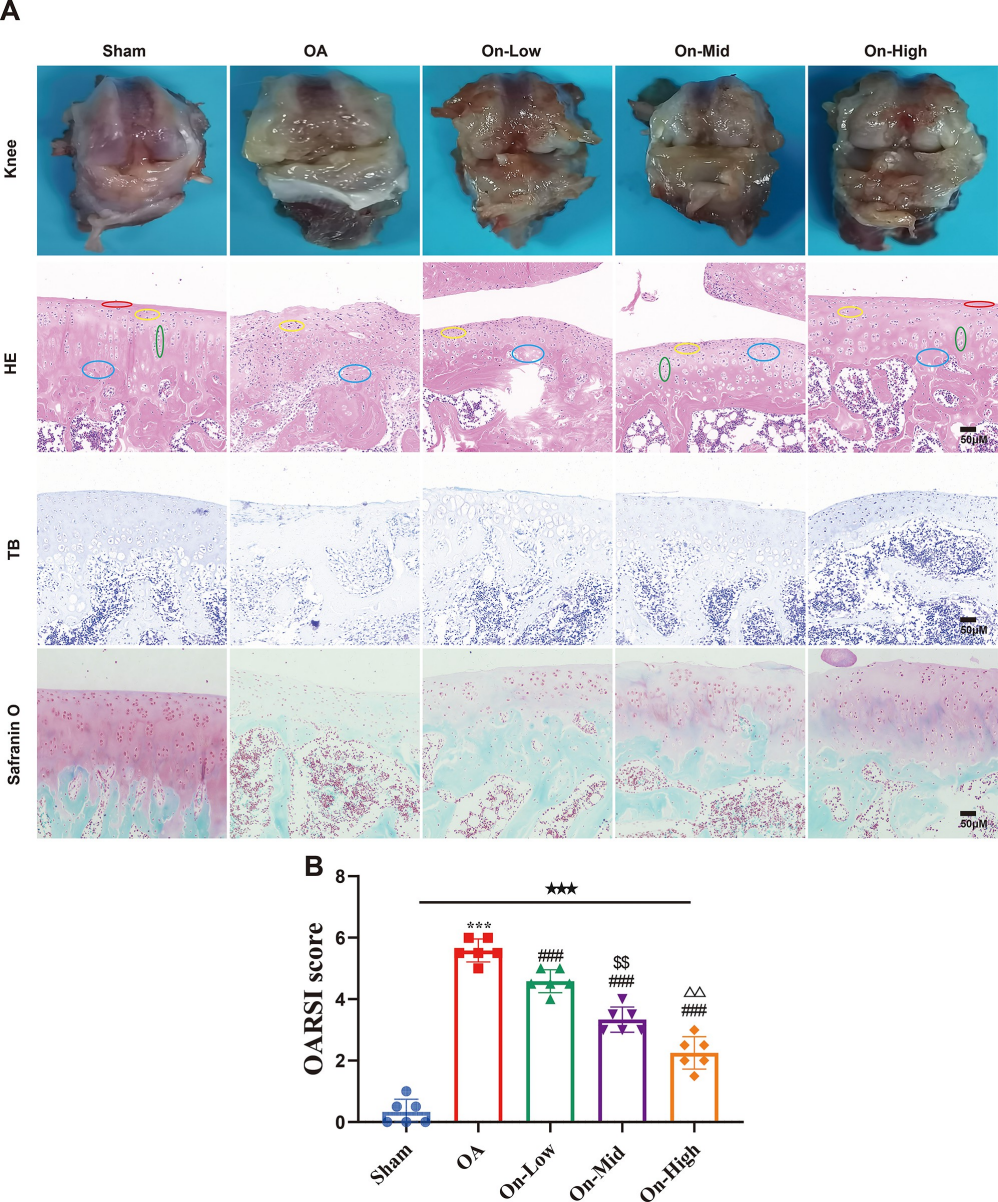
Ononin alleviated cartilage damage in OA rats. Representative pictures of (A) macroscopic observation and (histological staining of HE, TB and SF (scale bar: 50 μm) and (B) Osteoarthritis Research Society International (OARSI) scores of the femoral, n = 6. All data were presented as mean ± standard deviation. ***P < 0.001 vs. Sham group; ^###^P < 0.001 vs. OA group; ^$ $^P < 0.01 vs. On-Low group; ^△△^P< 0.01 vs. On-Mid group. ^★★★^P < 0.001 among the three groups. Red circles represent “superficial” area, yellow circles represent “migratory” area, green circles represent “radial” area, and blue circles represent “cartilaginous matrix calcifications” area.

After an 8-week treatment with ononin in OA rats, we assessed its protective effect on OA cartilage damage through superficial observations, HE, TB and SF staining assays. The gross morphology of the femoral condyles is depicted in Fig 4A. In the OA group, the articular cartilage exhibited rough surfaces, depressions, and cracks. Remarkably, after 8 weeks of treatment, ononin mitigated the aforementioned damage.

Subsequently, we conducted histological analysis of the femoral condyle cartilage from each group, as shown in Fig 4B. In the HE staining, cartilage nuclei appeared dark blue, while the cartilage ECM exhibited a pink hue. Additionally, the four layers of structure (superficial, migratory, radial, and cartilaginous matrix calcifications) were arranged regularly and clearly identifiable. However, in the OA group, the surface of the articular cartilage appeared rough, the four-layer structure was indistinct, and signs of fibrosis, degeneration, and cartilage defects were observed. Conversely, after treatment with ononin, the morphological structure showed improvement compared to the OA group. Regarding TB staining, cartilage nuclei appeared blue, acid glycosaminoglycans in the cartilage matrix appeared purple-blue and the subchondral bone matrix exhibited bright staining. Following treatment with ononin, the morphological structure of OA rats showed signs of repair.

Furthermore, in the SF staining, acid glycosaminoglycans in the cartilage matrix appeared bright red, while the subchondral bone exhibited green staining. In the OA group, the staining range of solid green was extended indicating a reduction in the content of glycosaminoglycan in cartilage matrix, an increase in fiber composition, and destruction of the cartilage. Conversely, the morphological structure of the ononin-treated groups showed signs of repair. Lastly, OARSI scores was used to quantify the severity of cartilage loss among different groups (Table 3 in S1 Raw data). The OARSI scores were inversely proportional, suggesting a protective effect against cartilage loss (Fig 4B).

### Ononin inhibited the secretion of inflammatory cytokines in OA rats

**Fig 5:**
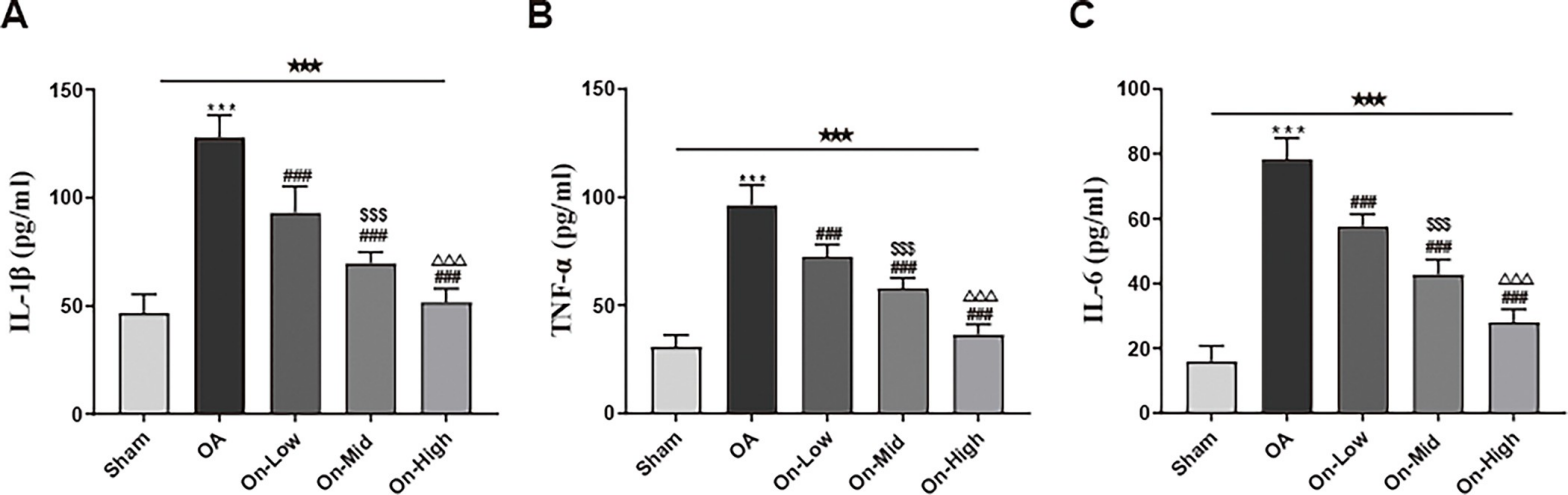
Ononin reduced the increase levels of IL-1β, TNF-α and IL-6 in OA rat serums. The levels of IL-1β (A), TNF-α (B) and IL-6 (C) inflammatory cytokines were accessed by ELISA, n = 10. All data were presented as mean ± standard deviation. ***P < 0.001 vs. Sham group; ^###^P < 0.001 vs. OA group; ^$ $ $^P < 0.001 vs. On-Low group; ^△△△^P < 0.001 vs. On-Mid group; ^★★★^P < 0.001 among the three groups.

Classical inflammatory cytokines such as IL-1β, Tumor necrosis factor-α (TNF-α) and Interleukin-6 (IL-6) act main role on development of OA [9]. We assayed the concentration of these factors in rats’ serum from each group to verify the anti-inflammatory function of ononin on OA (Tables 3–5 in S2 Raw data). The concentration of above factors in OA group were increased (P <0.001, Fig 5). After 8-week ononin treatment, this phenomenon can be reversed in a dose-dependent manner (P <0.05, Table 4 in S1 Raw data).

### Effect of ononin on expression of collagen II and MMP-13 in OA cartilage

**Fig 6:**
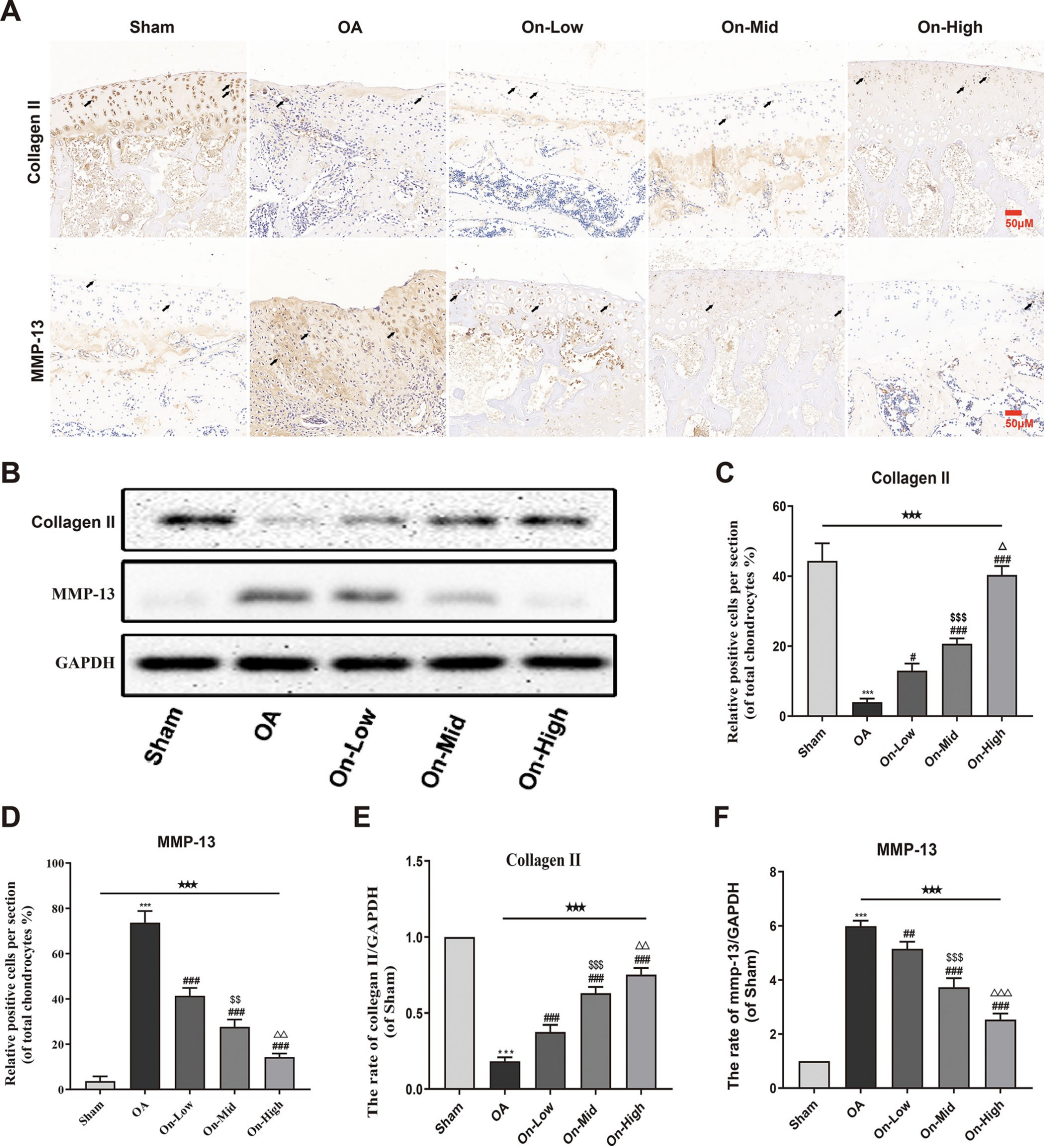
Effect of ononin on expression of collagen II and matrix metalloproteinase 13 (MMP-13) in OA cartilage. (A) Immunohistochemical staining was used to detect collagen II and MMP-13 levels in cartilage tissue (scale bar: 50 μm). Quantitative analysis of relative positive cells collagen II (C) and MMP13 (D) in the cartilage samples, n = 3. (B) Protein levels of collagen II and MMP-13 were determined by western blotting. Relative protein expressions of collagen II (E) and MMP-13 (F) was qualified by Image-J software, n = 4. All data were presented as mean ± standard deviation. ***P < 0.001 vs. Sham group; ^#^P < 0.05 vs. OA group; ^##^P < 0.01 vs. OA group; ^###^P < 0.001 vs. OA group; ^$ $^P < 0.01 vs. On-Low group; ^$ $ $^P < 0.001 vs. On-Low group; ^△^P < 0.05 vs. On-Mid group; ^△△^P < 0.01 vs. On-Mid group; ^△△△^P < 0.001 vs. On-Mid group; ^★★★^P < 0.001 among the three groups. The positive cells with black arrows.

To quantitatively assess cartilage degradation, we analyzed the expression of collagen II and MMP-13 using immunohistochemistry and Western blot methods (Fig 6A and 6B, Figs 1–3 in S1 Raw images). Immunohistochemistry revealed that the ECM of OA rats’ cartilage exhibited decomposition, as evidenced by changing the levels of these proteins. However, ononin treatment reversed this damage, as observed compared to the Sham group (Fig 6C and 6D, Table 5 in S1 Raw data). Consistently, similar results were obtained using Western bolt (P<0.001, Fig 6E and 6F, Table 6 in S1 Raw data). In summary, these results confirm that ononin can attenuate the degradation of cartilage ECM (Tables 6–9 in S2 Raw data), suggesting its potential therapeutic efficacy in osteoarthritis.

### Ononin repressed the reaction of MAPK and NF-κB pathways in OA rats

**Fig 7:**
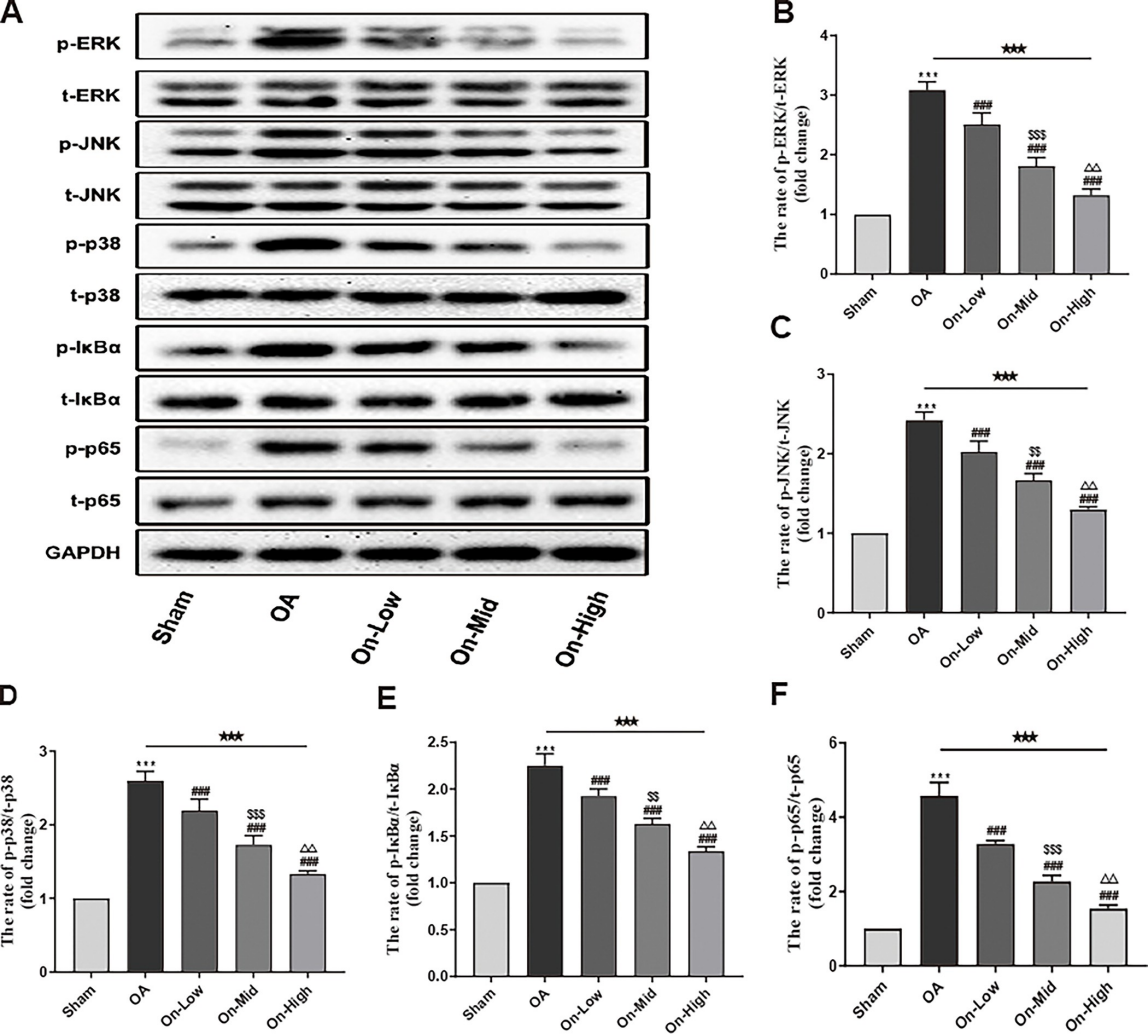
Ononin repressed the activation of MAPK and NF-κB signaling pathways in OA rats. (A) The phosphorylation and total proteins were determined by western blotting. Relative phosphorylation levels of proteins were qualified by ImageJ software and was normalized by corresponding total protein content, n = 4. All data were presented as mean ± standard deviation. ***P < 0.001 vs. Sham group; ^###^P < 0.001 vs. OA group; ^$ $^P < 0.01 vs. On-Low group; ^$ $ $^P < 0.001 vs. On-Low group; ^△△^P < 0.01 vs. On-Mid group; ^★★★^P < 0.001 among the three groups.

**Fig 8:**
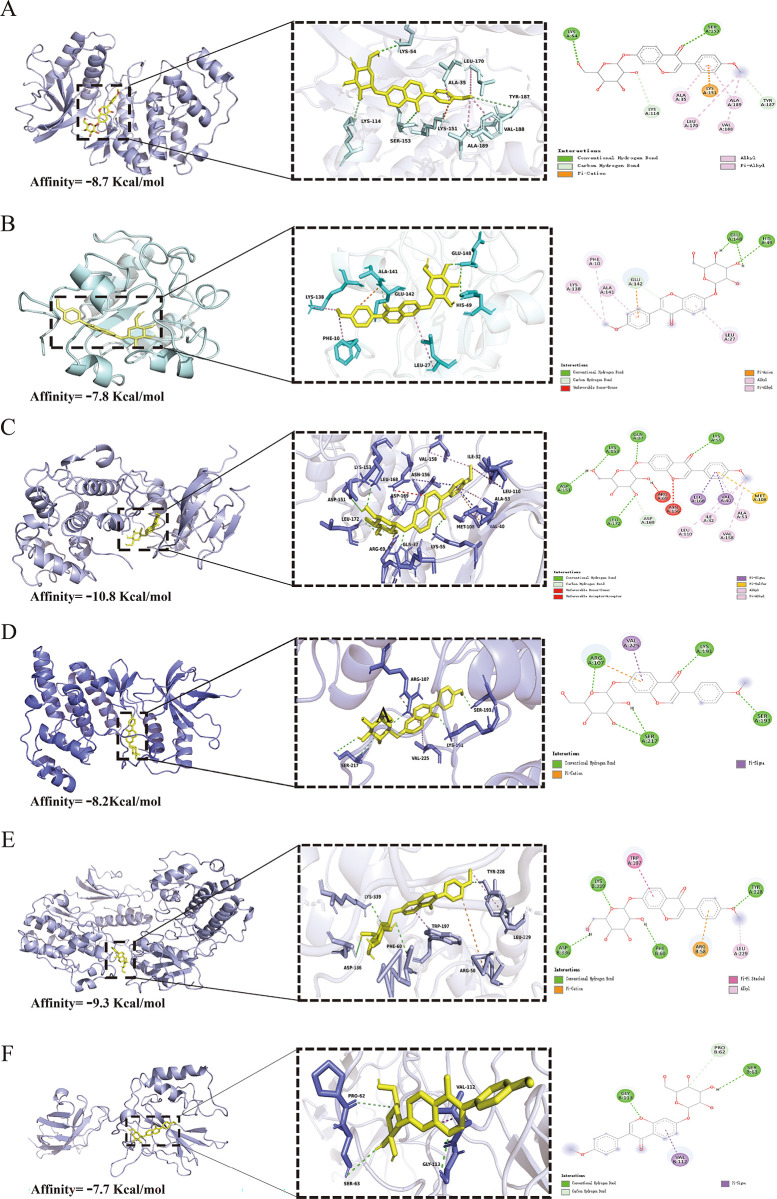
The results of molecular docking between the key ingredients (ligands) and core targets (receptors). (A) The binding effect of ononin and ERK1 (affinity: −8.7 Kcal/mol). (B) The binding effect of ononin and ERK2 (affinity: −7.8 Kcal/mol). (C) The binding effect of ononin and JNK1 (affinity: −10.8 Kcal/mol). (D) The binding effect of ononin and JNK2 (affinity: −8.2 Kcal/mol). (E) The binding effect of ononin and p38 (affinity: −9.3 Kcal/mol). (F) The binding effect of ononin and p65 (affinity: −7.7 Kcal/mol).

We quantified the phosphorylation levels of ERK, JNK, p38, IκBα, and p65 in cartilage tissues of OA rats to investigate the regulatory effects of ononin on the MAPK and NF-κB pathways (Tables 10–14 in S2 Raw data). Upon testing, compared with the Sham group, the phosphorylated proteins mentioned above were significantly increased in the articular cartilages of rats in the OA group (Figs 4–14 in S1 Raw images). Remarkably, ononin treatment suppressed the phosphorylation levels of proteins within the MAPK and NF-κB pathways (P<0.05, Fig 7, Table 7 in S1 Raw data).

### Docking analysis

As shown in Fig 8, the Vina script was employed to dock the receptor proteins ERK1, ERK2, JNK1, JNK2, p38, and p65, resulting in binding energies were −8.7, -7.8, -10.8, -8.2, -9.3, and −7.7 kcal/mol, respectively. These values indicate that the ligand molecules could stably bind to the receptor proteins. Subsequently, PyMOL was used to analyze and compare the interactions of the ligand-receptor protein complexes. Ononin forms hydrogen bonds with LYS54 and SER153 of the ERK1 receptor protein. It forms hydrogen bonds with GLU148 and HIS49 of ERK2. For the receptor protein JNK1, ononin forms hydrogen bonds with ASP151, LYS153, LEU172, and GLN37. With the receptor protein JNK2, ononin forms hydrogen bonds with ARG107, LYS191, and SER217. For the receptor protein p38, ononin forms hydrogen bonds with ASP336, LYS339, PHE60, and TYR228. Lastly, with the receptor protein p65, ononin forms hydrogen bonds with GLN113 and SER63. These interactions facilitate stable binding of small molecules and proteins within their pockets.

## Discussion

OA is characterized by cartilage degeneration and is often accompanied by symptoms such as inflammation, pain, joint structural pathology, and loss of function, which significantly inconveniencing patients’ daily life [26]. To date, underlying the pathological changes in OA are not fully understood, and effective therapies remain largely unavailable [27]. Recently, Chinese herbal compounds have garnered significant attention for OA therapy due to their anti-inflammatory and antioxidant activities [18, 28]. Notably, isoflavone compounds present in legumes have been shown to protect cartilage in OA treatment [19]. The efficacy of ononin, an isoflavone compound, has been documented in models of Alzheimer’s disease [23], LPS-induced zebrafish inflammation [29], and rheumatoid arthritis [30]. Additionally, our study found that ononin treatment exhibited minimal side effects.

Our study is the first to report that ononin exerts anti-inflammatory effects in the ACLT-induced OA rat model. The OA’s pathological manifestations include chondrocyte loss and cartilage ECM degradation [31, 32]. In this research, we observed a significant increase in the number of cartilage cells, as evidenced by TB staining, and a reduction of OARSI scores in ononin-treated OA rats, indicating a profound reversal in cartilage damage. This was accompanied by a reduction in MMP-13 protein levels and up-regulation of collagen II. Moreover, ononin suppressed the secretion of inflammatory cytokines in OA rats. Additionally, ononin inhibited the abnormal phosphorylation of ERK, JNK, p38, IκBα and p65. In summary, ononin may ameliorate OA cartilage damage by down-regulating the MAPK and NF-κB inflammation-dependent pathways. Therefore, ononin represents a promising and beneficial treatment for OA.

The pathological development of OA is closely linked to the inflammatory process. OA cartilage is infiltrated with various cytokines and proteases that contribute to the destruction of the ECM [33, 34]. Elevated levels of inflammatory cytokines in the knee cartilage tissue of OA patients are considered a major feature of OA [35, 36]. Previous research has demonstrated that alpinia oxyphylla inhibited the secretion of inflammatory factors and mediators (prostaglandin E2 and nitric oxide), thereby reducing joint pain and attenuating cartilage degradation [37]. Similarly, daidzein has been shown to reduce the levels of inflammatory factors in an OA model [38]. In this study, ononin, a Chinese herbal compound, exhibited similar effect in OA rats. After an 8-week treatment with ononin, our pathological results and OARSI score supported these findings. Ononin alleviated cartilage damage by inhibiting the inflammatory response, thereby demonstrating its potential as an effective treatment for OA.

Data suggest that cartilage loss in OA is associated with the abnormal high expression of inflammatory mediators, stress response factors, and cartilage-degrading proteases [39]. Therefore, inhibiting the secretion of inflammatory mediators and cartilage-degrading proteases to prevent ECM degradation in OA cartilage has become a standard practice in OA treatment [40]. During the pathological development of OA, MMPs play a critical role in degrading ECM proteins, leading to articular cartilage damage. The production of MMPs, particularly MMP-13, can be significantly increased by pro-inflammatory cytokines [41–43]. OA is characterized by elevated levels of MMP-13 in cartilage, which contributes to ECM degradation [44]. Additionally, fragments of degraded collagen II can initiate inflammatory response, resulting in the release of more inflammatory mediators that further aggravate cartilage degradation [45]. This creates a vicious cycle of abnormal inflammatory response and ECM degradation, necessitating timely drug intervention for effective treatment for effective treatment. Evidence indicates that resveratrol delays joint degeneration by reducing the activity of MMP-13 in OA modals [46]. In our study, ononin inhibited the expression of MMP-13 while increasing the expression of collagen II in the cartilage of OA rats (Fig 6). Thus, it is likely that ononin repaired cartilage damage by modulating these two proteins, thereby disrupting the cycle of inflammation and degradation.

Furthermore, there is substantial evidence suggesting that the MAPK and NF-κB pathways are associated with inflammatory reactions, chondrocyte apoptosis, calcification, metabolism, and the synthesis of MMPs in the OA development [47, 48]. The MAPK pathway can be activated by various inflammatory factors; one study found that TNF-α can activate the MAPK pathway, exacerbating chondrocyte proteoglycan degradation and increasing the secretion of MMPs in knee arthritis [49, 50]. Inflammatory factors such as TNF-α can activate IKK, which in turn phosphorylating IκBα, leading to the dissociation of the intracellular NF-κB·IκB complex. This dissociation allows NF-κB to translocate into the nucleus and initiate the production of inflammatory mediators [51, 52]. Previous studies have shown that curcumol blocked the activities of these pathways, thereby alleviating the inflammatory response and ECM destabilization in OA [53]. Our study confirmed that the phosphorylation levels of ERK, JNK, p38, IκBα and p65 were increased in cartilage of OA rats. However, treatment with different doses of ononin effectively inhibited the phosphorylation of these proteins. Moreover, our molecular docking results corroborate the tight interaction of ononin with key proteins of the signalling pathway.

Moreover, our molecular docking results corroborate the tight interaction of ononin with key proteins in the signaling pathway. The binding energies obtained from the docking studies indicate stable interactions between ononin and the receptor proteins ERK1, ERK2, JNK1, JNK2, P38, and P65. These interactions suggest that ononin can effectively bind to and potentially inhibit the activity of these proteins, thereby modulating the MAPK and NF-κB pathways. This molecular interaction supports the observed biochemical effects of ononin in reducing the phosphorylation levels of ERK, JNK, p38, IκBα, and p65, further validating its potential as a therapeutic agent for OA. These findings suggest that ononin may exert its therapeutic effects in OA by targeting and modulating the MAPK and NF-κB pathways, thereby reducing inflammation and preventing ECM degradation.

Current conservative treatment strategies for OA primarily rely on the use of NSAIDs [16, 54]. However, the long-term use of NSAIDs is limited due to their side effects on the gastrointestinal system, imposing both physical and economic burden to patients and failing to restore articular cartilage [17]. Consequently, there is an urgent need to develop novel drugs, such as those derived from Chinese herbs, which have limited side effects for the treatment of OA. As discussed, the anti-inflammatory effects of ononin have been verified in OA models. This research demonstrated that ononin decreases the levels of inflammatory cytokines and regulates the MAPK and NF-κB pathways, indicating its potential clinical benefit in the treatment of OA.

## Conclusion

In conclusion, our research demonstrated that ononin improved cartilage damage in ACLT-induced OA rats by reducing the levels of IL-1β, TNF-α and IL-6 inflammatory factors and recovering the ECM of the cartilage. Additionally, ononin inhibited the phosphorylation of ERK, JNK, p38, IκBα and p65 proteins. Overall, ononin was able to alleviate inflammation and repair cartilage loss by regulating the MAPK and NF-κB pathways in the OA rat model. However, further studies are needed to determine whether ononin can delay the overall progression of OA.

## Supporting information

S1 Raw images (DOCX)

S1 Raw data (DOCX)

S2 Raw data (XLSX)
