# Supplementary material for: Ononin delays the development of osteoarthritis by down-regulating MAPK and NF-κB pathways in rat models
Source: PLoS One. 2024 Oct 31;19(10):e0310293. doi: 10.1371/journal.pone.0310293 (PMC11527302; doi:10.1371/journal.pone.0310293)
Supplement: S1 Raw images — (DOCX) [file pone.0310293.s001.docx]

**Supplementary Information**

**the full length original blot**

**Collegan II:**


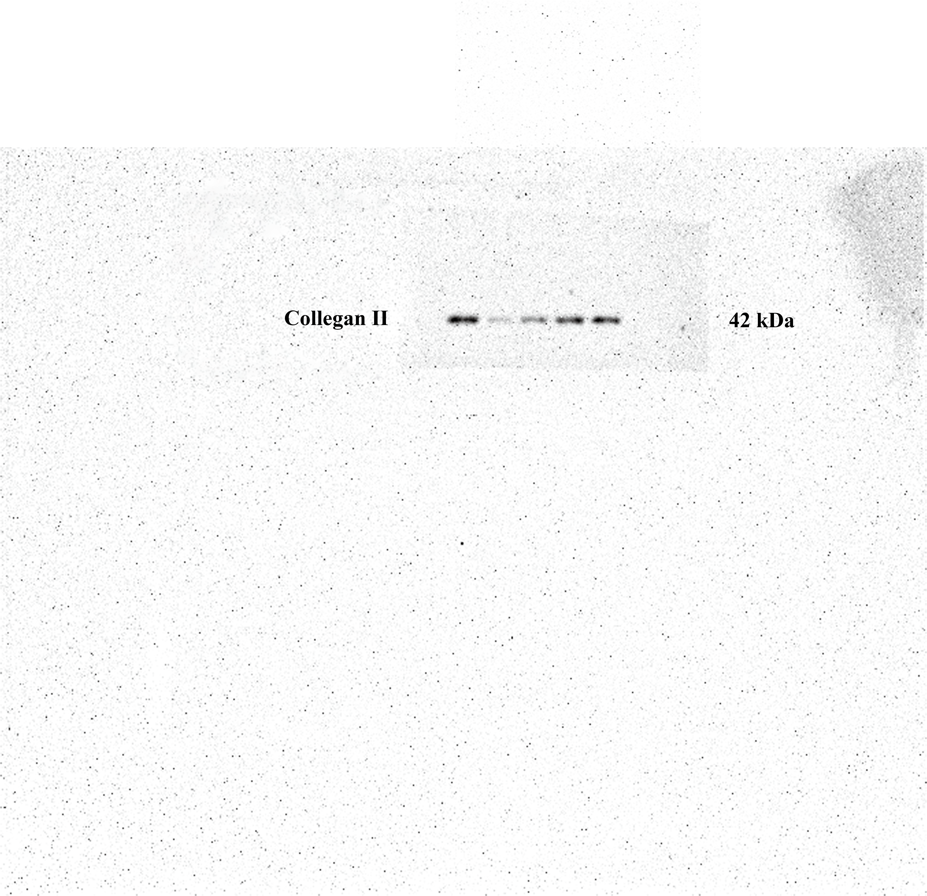


**Fig.1 the original blot of collegan II.**

**MMP-13:**

**
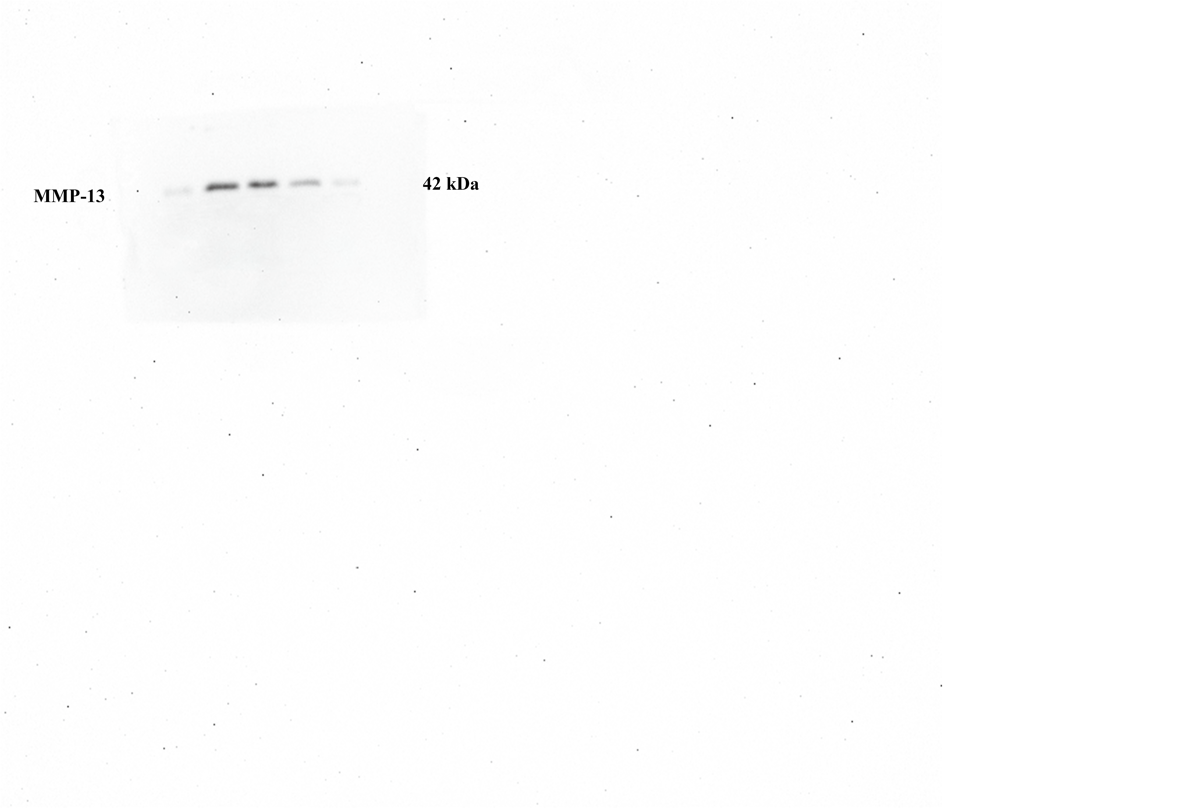
**

**Fig.2 the original blot of MMP-13.**

**GAPDH (for collegan II and MMP-13 proteins):**

**
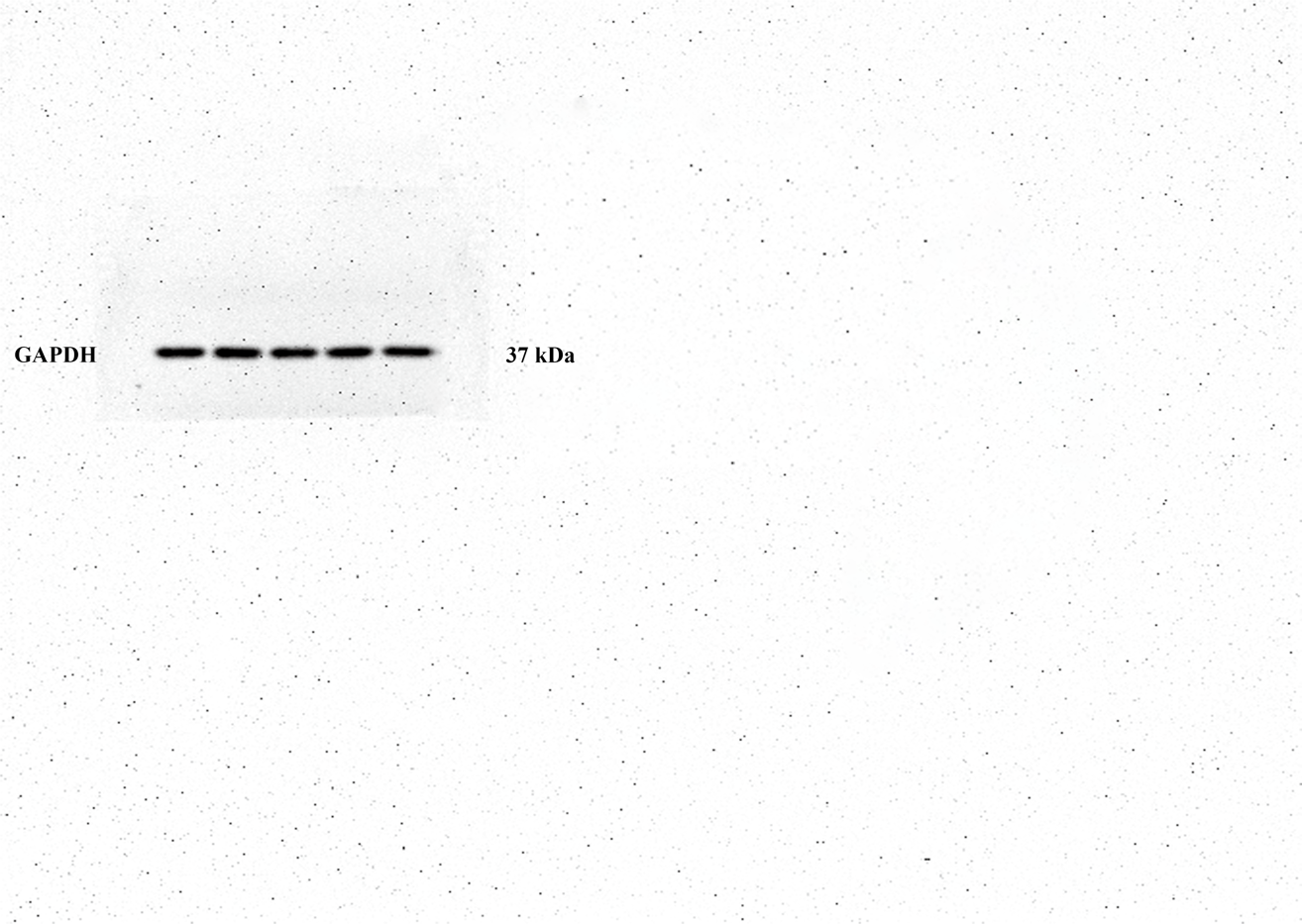
**

**Fig.3 the original blot of GAPDH (for collegan II and MMP-13 proteins).**

**p-ERK:**

**
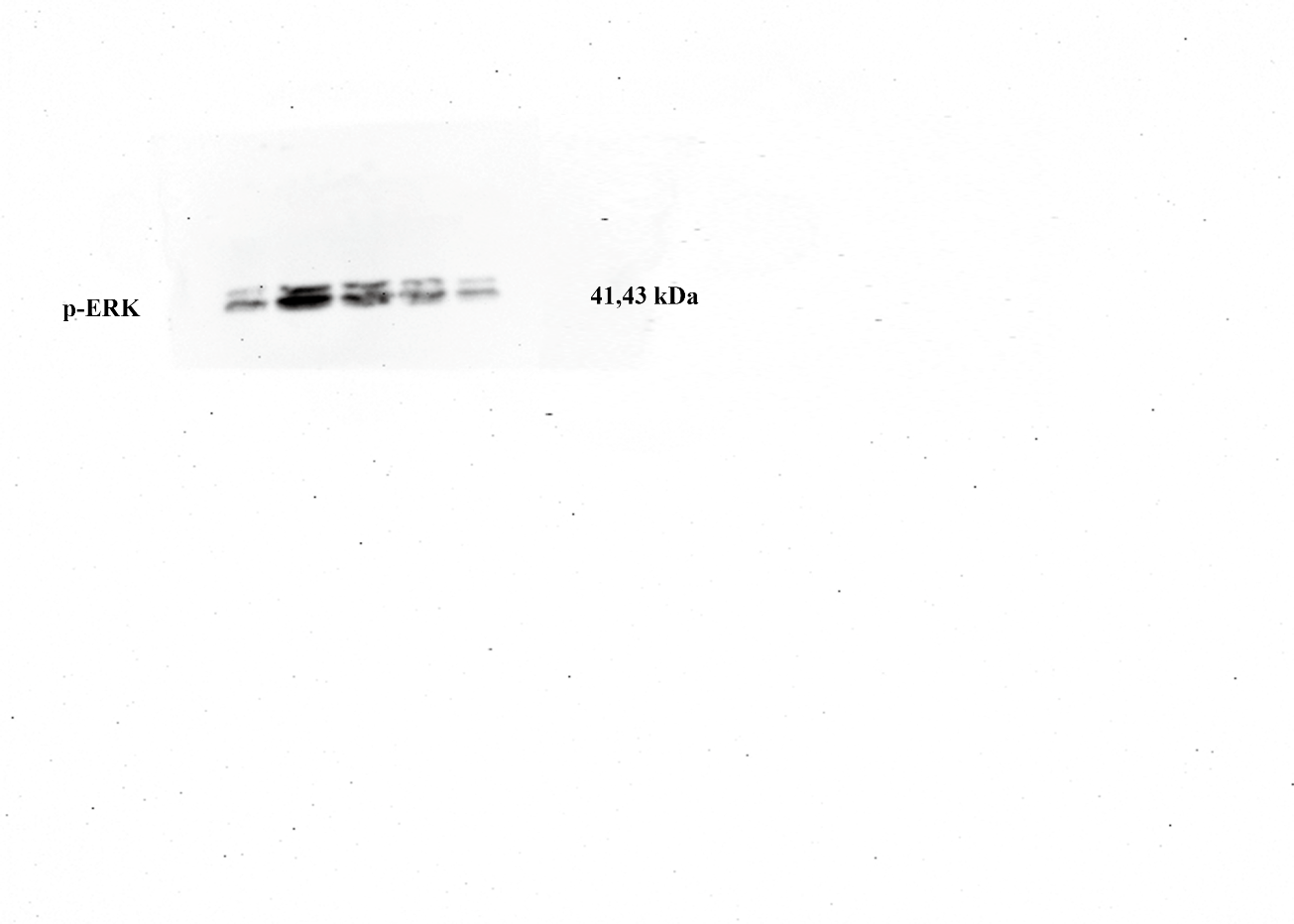
**

**Fig.4 the original blot of p-ERK.**

**t-ERK:**

**
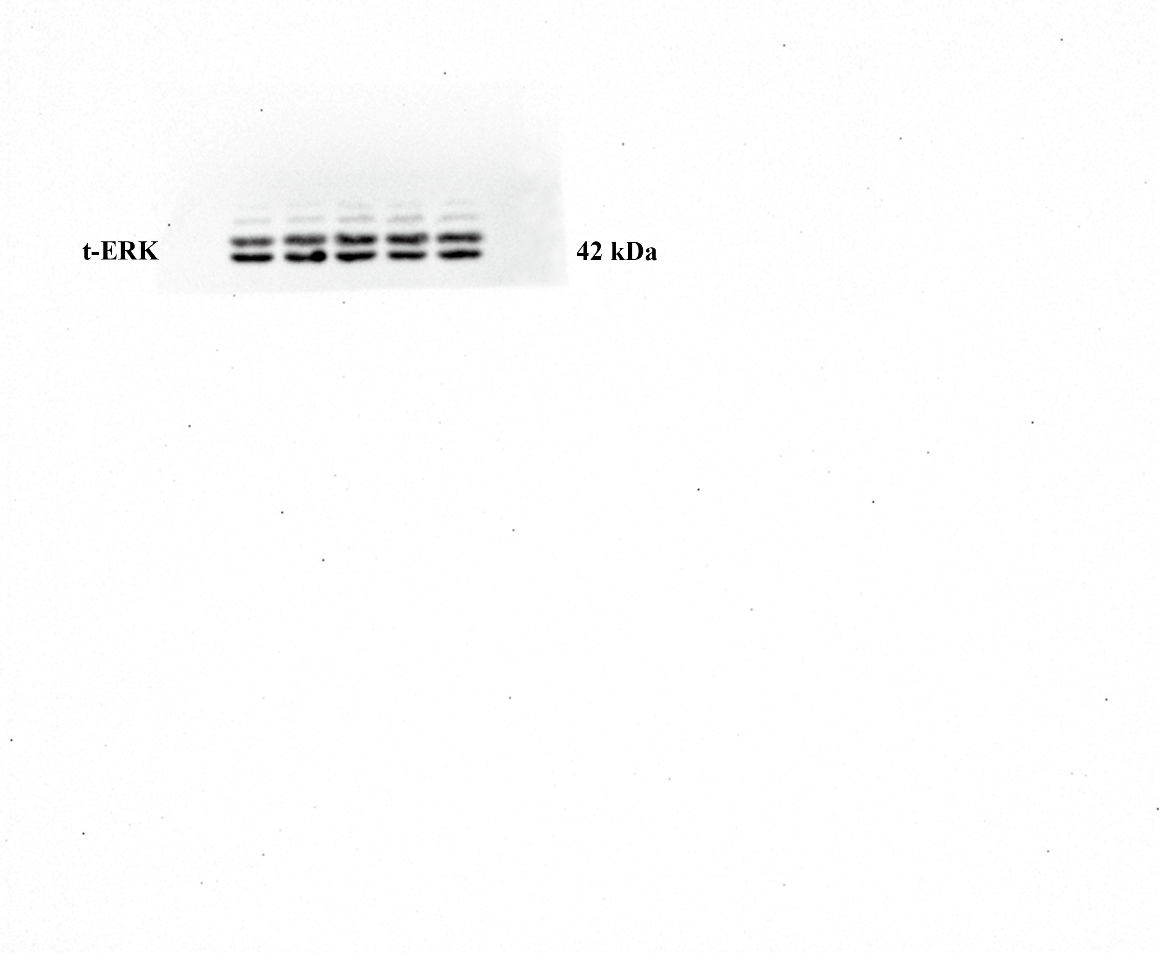
**

**Fig.5 the original blot of t-ERK.**

**p-JNK:**

**
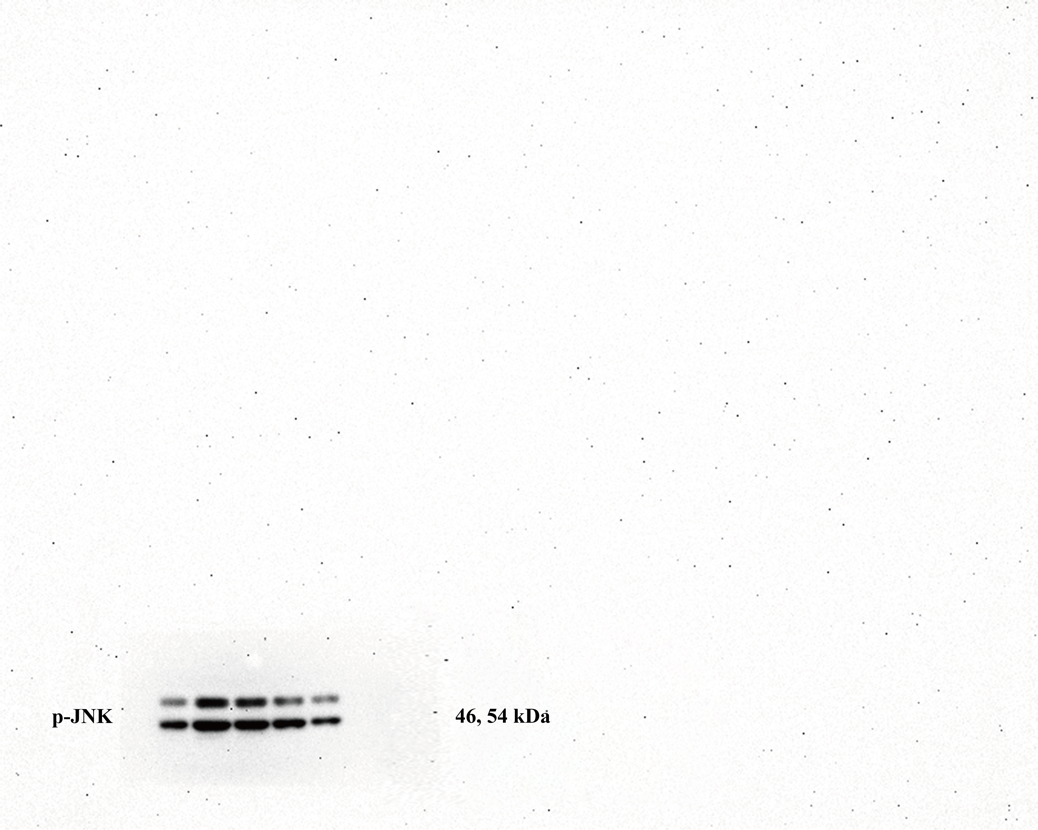
**

**Fig.6 the original blot of p-JNK.**

**t-JNK:**

**
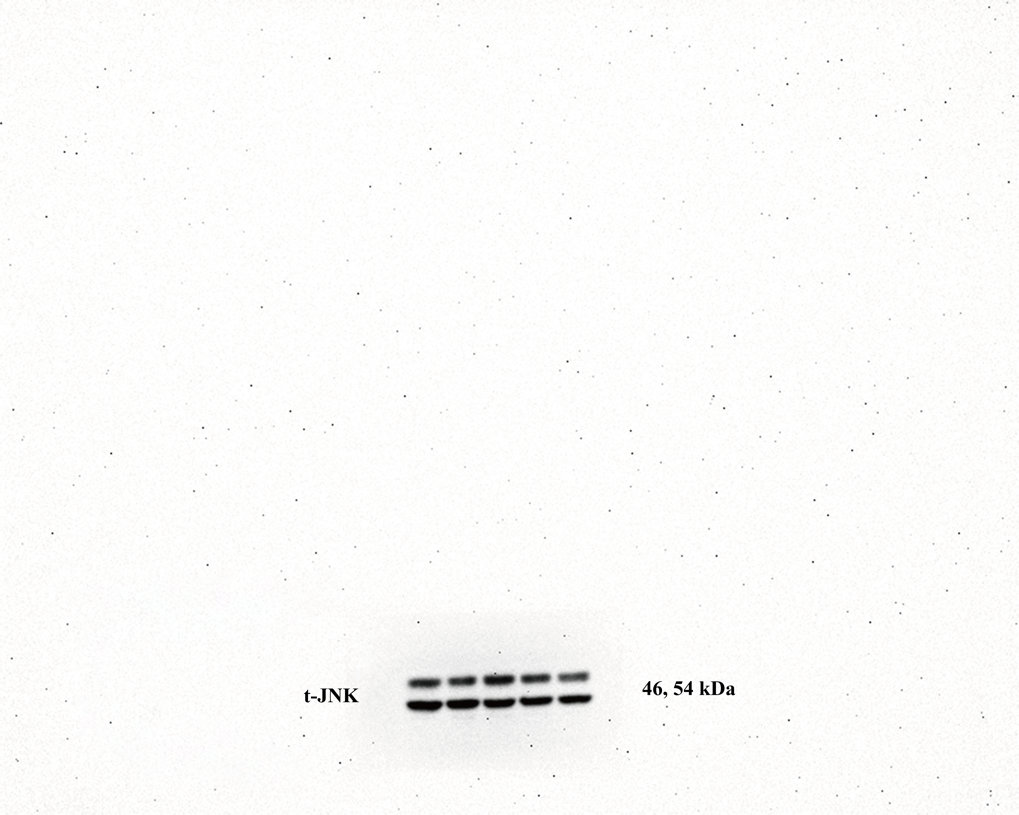
**

**Fig.7 the original blot of t-JNK.**

**p-P38:**

**
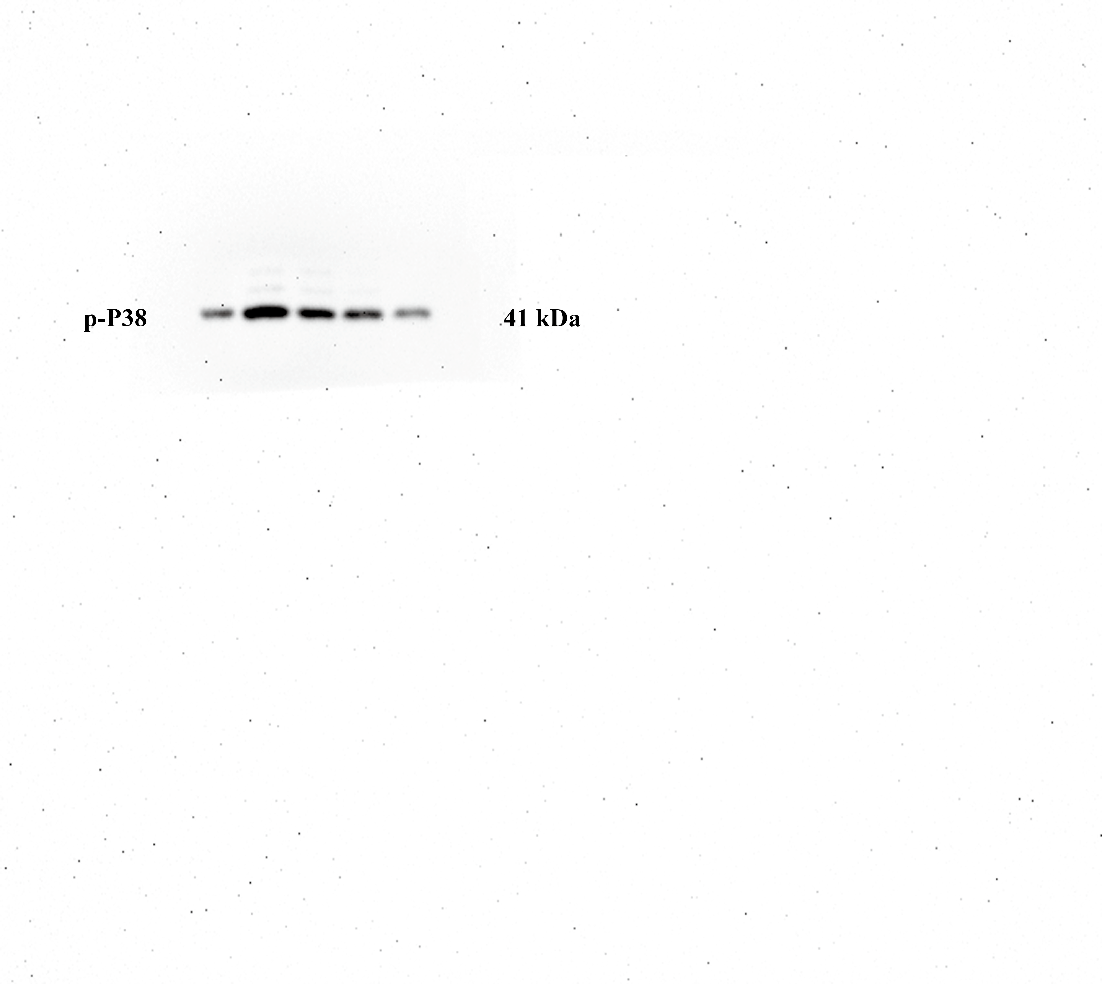
**

**Fig.8 the original blot of p-P38.**

**t-P38:**

**
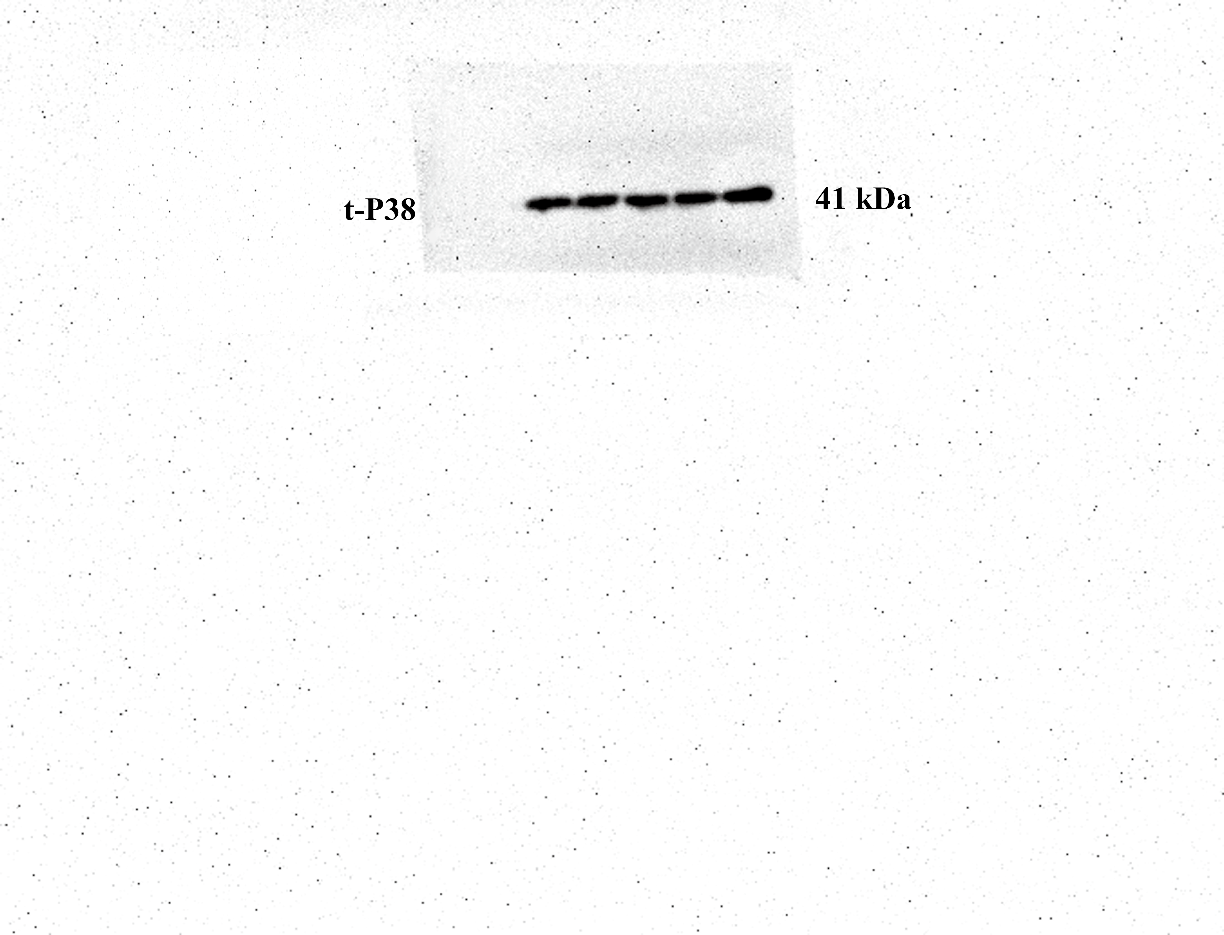
**

**Fig.9 the original blot of t-P38.**

**p-IκBα:**

**
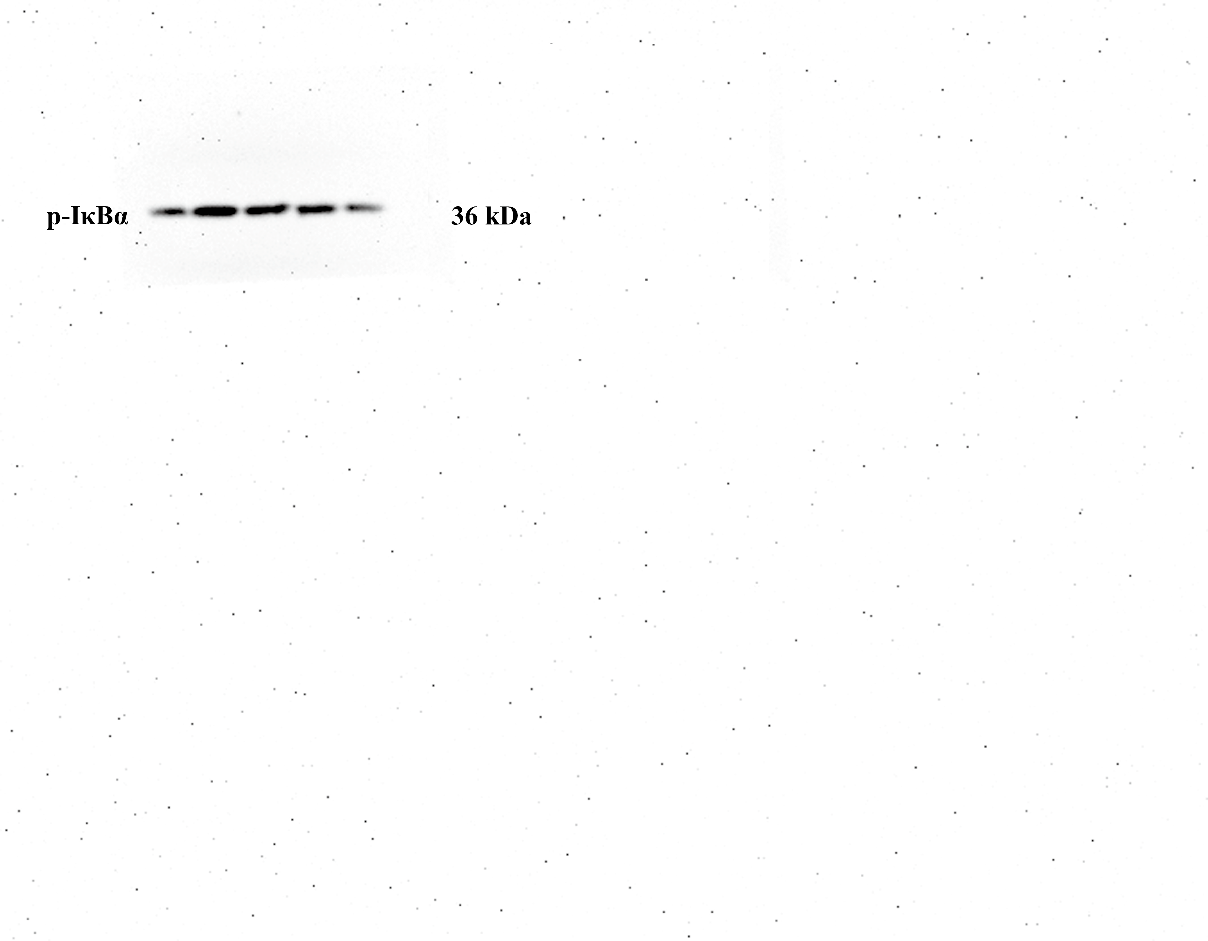
**

**Fig.10 the original blot of p-IκBα.**

**t-IκBα:**

**
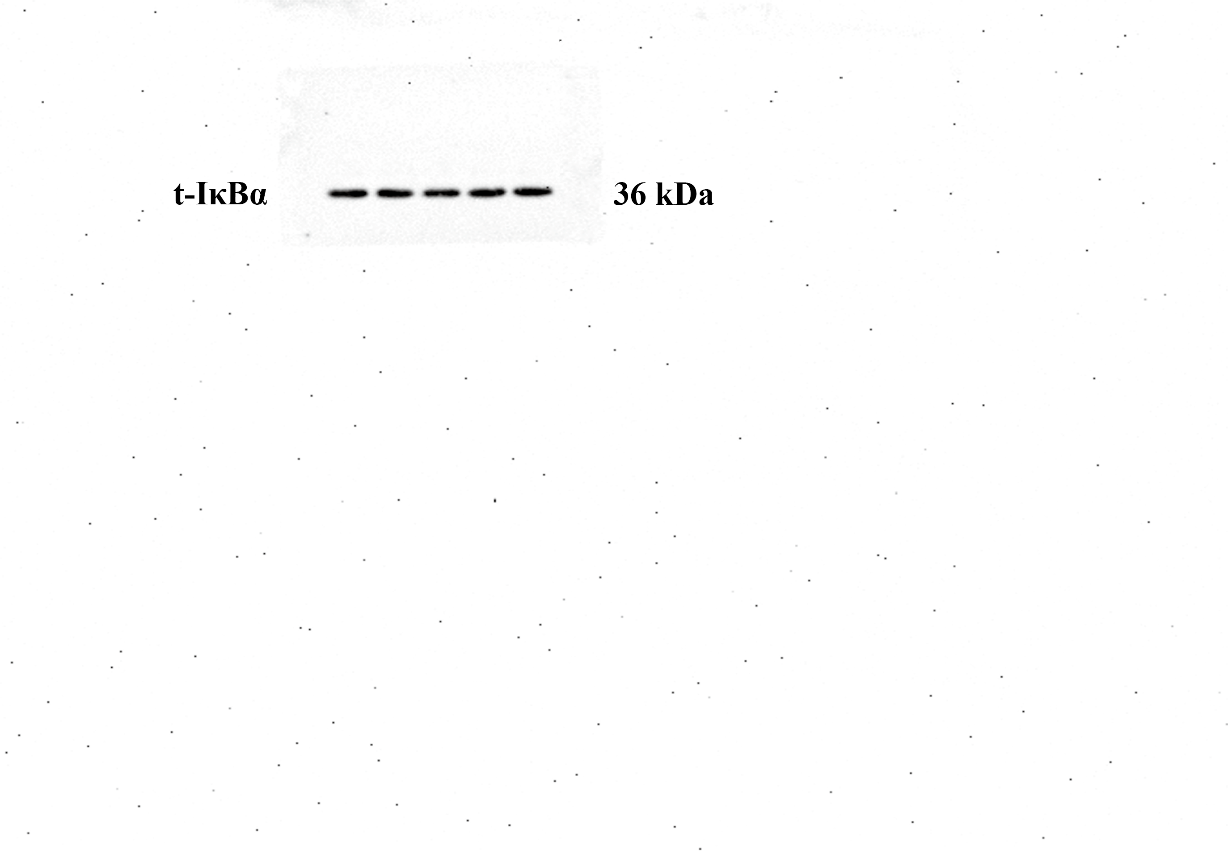
**

**Fig.11 the original blot of t-IκBα.**

**p-P65 (upmost blot):**

**
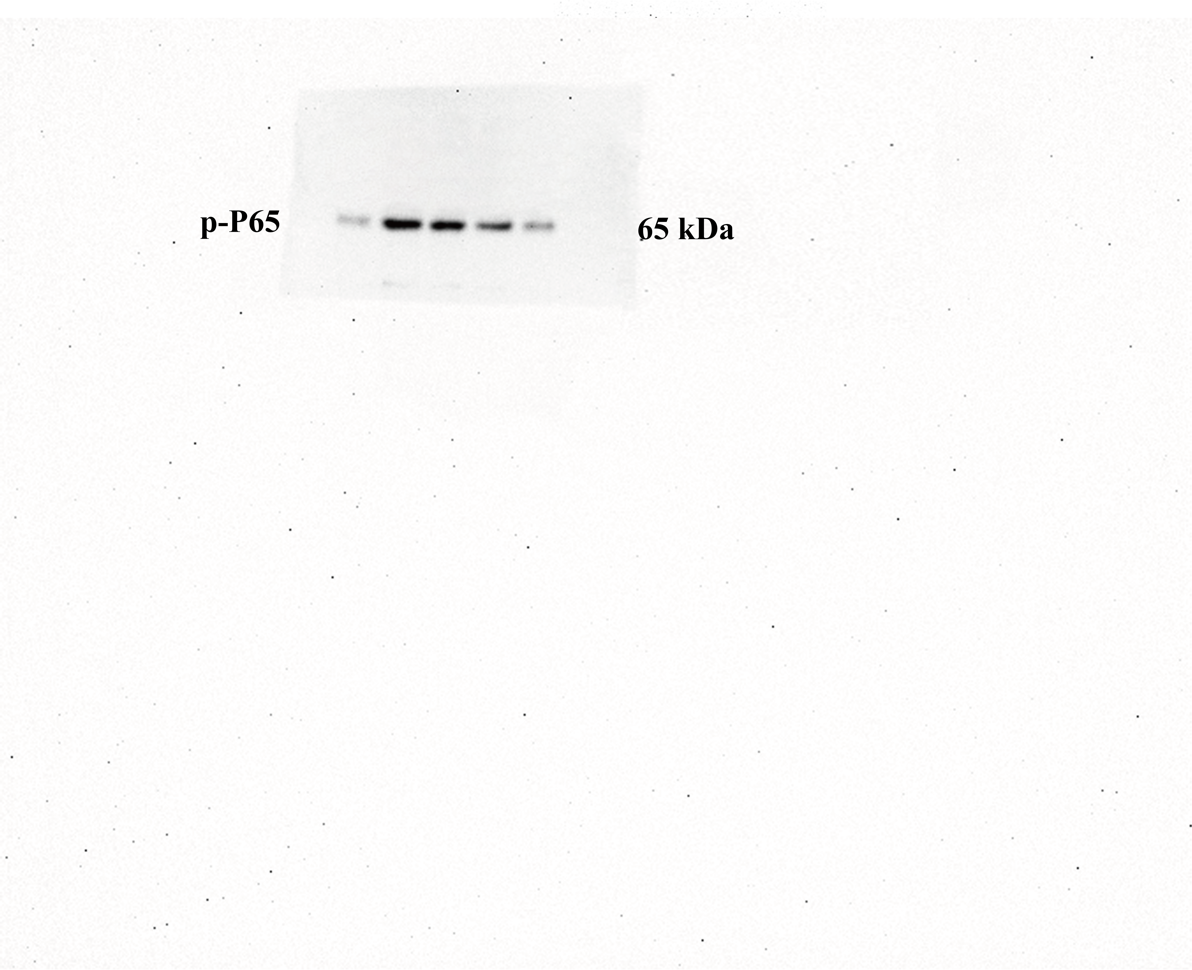
**

**Fig.12 the original blot of p-P65.**

**t-P65** (**the top of the blot):**

**
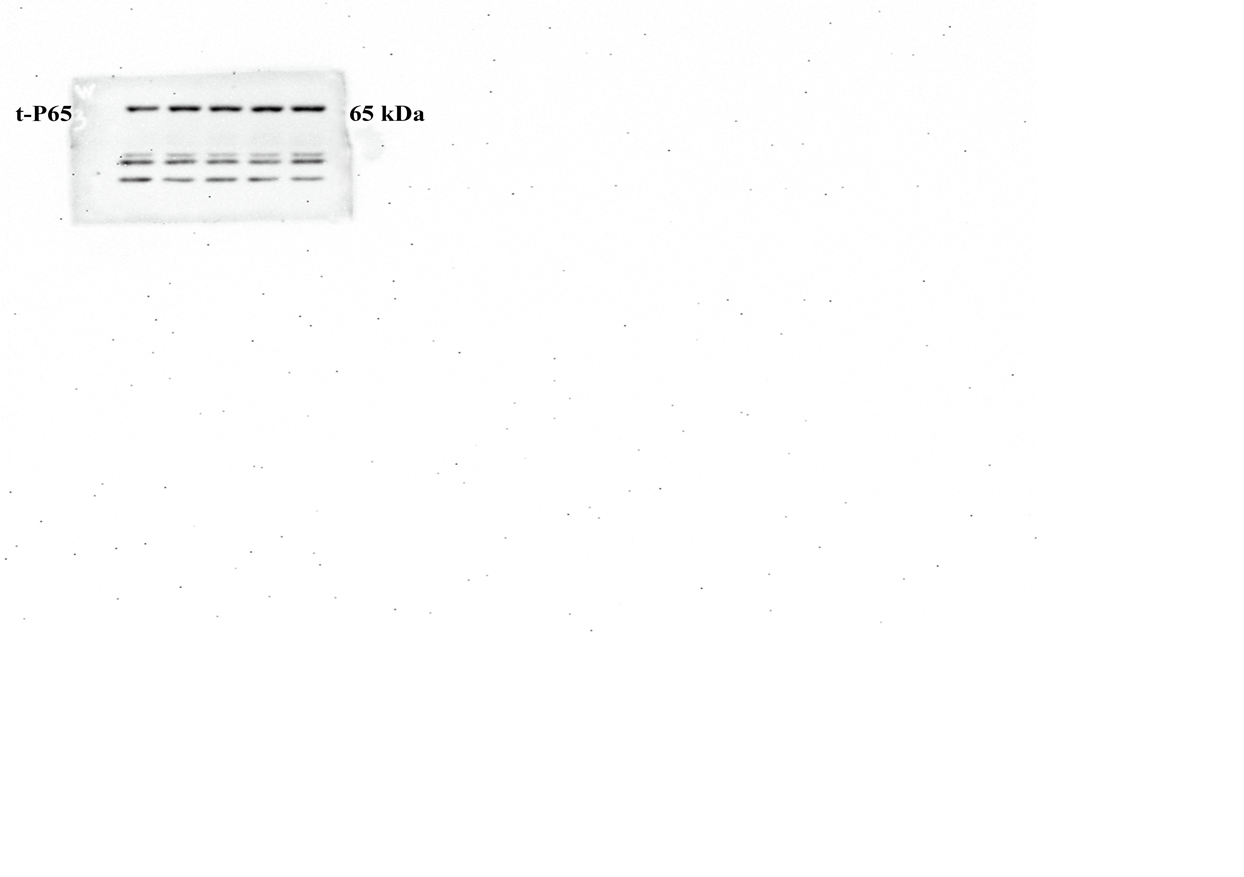
**

**Fig.13 the original blot of t-P65** (**the top of the blot).**

**GAPDH (for p-ERK/t-ERK, p-JNK/t-JNK, p-P38/t-P38, p-IκBα/t-IκBα and p-P65/t-P65 proteins):**

**
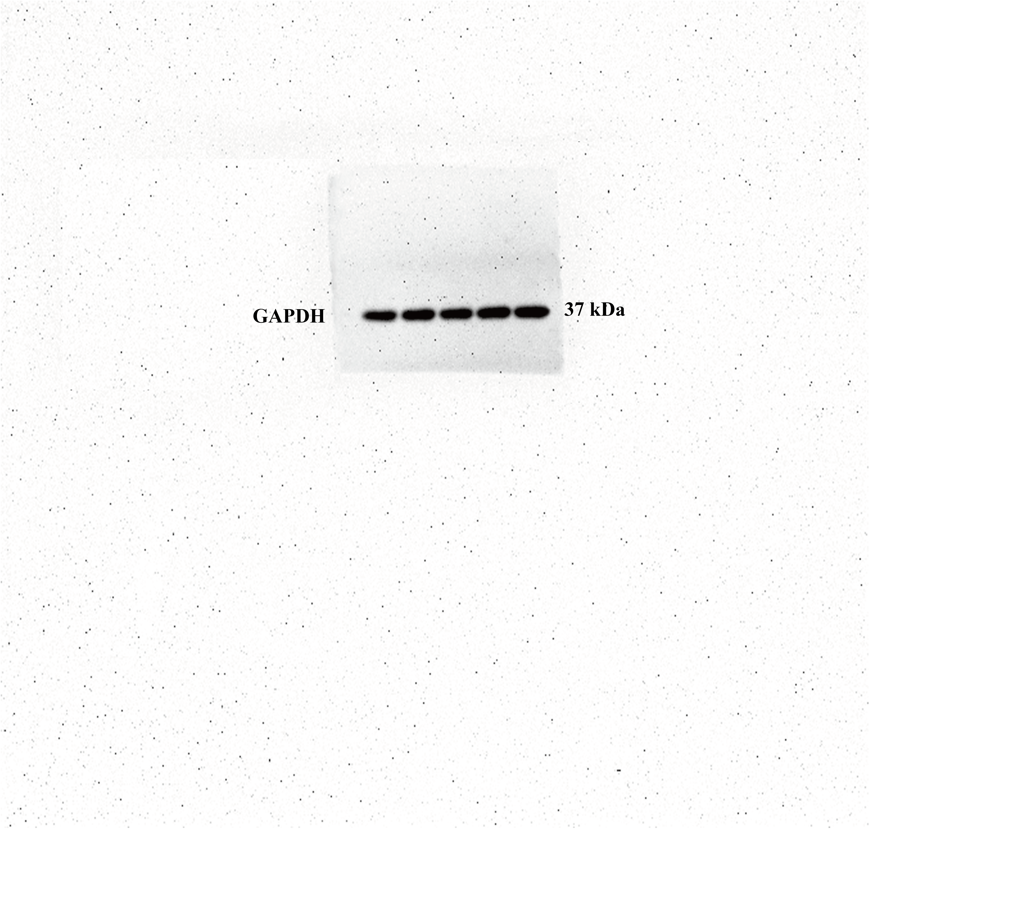
**

**Fig.14 the original blot of GAPDH (for p-ERK/t-ERK, p-JNK/t-JNK, p-P38/t-P38, p-IκBα/t-IκBα and p-P65/t-P65 proteins).**
