# Supplementary material for: Ononin delays the development of osteoarthritis by down-regulating MAPK and NF-κB pathways in rat models
Source: PLoS One. 2024 Oct 31;19(10):e0310293. doi: 10.1371/journal.pone.0310293 (PMC11527302; doi:10.1371/journal.pone.0310293)
Supplement: S1 Raw data — (DOCX) [file pone.0310293.s002.docx]

**S2 Raw data**

**Table 1.** Curve of body weight of rats over time (value, Mean ± SD).

| Groups | N | Fig 3B | | | | | | | | |  |
| --- | --- | --- | --- | --- | --- | --- | --- | --- | --- | --- | --- |
|  |  | 1^st^ week | 2^nd^ week | 3^rd^ week | 4^th^ week | 5^th^ week | 6^th^ week | 7^th^ week | 8^th^ week | 9^th^ week |  |
| Sham | | 10 | 203.2±4.78 | 226.7±6.02 | 268.7±5.27 | 295.1±4.91 | 309.9±5.49 | 330.3±4.88 | 346±5.29 | 364.3±4.99 | 375.8±6.21 |
| OA | | 10 | 202.7±12.75 | 216.3±8.81 | 260±8.65 | 288.6±11.66 | 313±12.91 | 322±10.22 | 336.7±11.44 | 360.1±10.25 | 378.1±9.85 |
| On-Low | | 10 | 199.2±6.73 | 222.8±8.87 | 262.9±8.9 | 284.9±8.32 | 307.5±11.54 | 320.6±8.34 | 334.8±8.28 | 359.1±11.4 | 370.63±7.72 |
| On-Mid | | 10 | 199±5.72 | 219.7±8.29 | 260.9±8.44 | 291.9±7.82 | 308±9.79 | 320.3±8.47 | 337.9±10.86 | 353.2±12.15 | 377.1±7.34 |
| On-High | | 10 | 202.5±8.4 | 225.6±11.1 | 266.8±11.27 | 288.6±10.22 | 312.9±13.79 | 322.8±14.31 | 337.6±13.1 | 358±10.8 | 380.5±7.21 |
| F value | |  | 0.6258 | 2.382 | 1.854 | 1.879 | 0.5548 | 1.77 | 1.796 | 1.525 | 0.4657 |
| P value | |  | 0.6466 | 0.0655 | 0.1352 | 0.1306 | 0.6965 | 0.1516 | 0.1462 | 0.2111 | 0.7605 |

Comparisons were made between 5 groups for each time period: P > 0.05, no significant differences from each group. The weights of all samples for each time period are shown in S3 Table 1.

**Table 2.** The organ coefficients of liver, spleen and kidney were measured after 8 weeks of post-surgery (value, Mean ± SD).

| Groups | N | Fig 3C | | |
| --- | --- | --- | --- | --- |
|  |  | Liver | Spleen | Kidney |
| Sham | 3 | 2.84 ± 0.07 | 0.62 ± 0.08 | 0.15 ± 0.05 |
| OA | 3 | 2.88 ± 0.28 | 0.64 ± 0.08 | 0.16 ± 0.04 |
| On-Low | 3 | 2.91 ± 0.14 | 0.61 ± 0.06 | 0.14 ± 0.04 |
| On-Mid | 3 | 3±0.12 | 0.63±0.06 | 0.14±0.03 |
| On-High | 3 | 2.81±0.2 | 0.6±0.03 | 0.15±0.03 |
| F value |  | 0.5083 | 0.2048 | 0.1807 |
| P value |  | 0.7312 | 0.9299 | 0.9432 |

Comparisons were made between 5 groups for organ coefficients of liver, spleen and kidney: P > 0.05, no significant differences from each group. Additional information on data in S3 Table 2.

**Table 3.** Osteoarthritis Research Society International (OARSI) scores of the femoral. (n=10, Mean ± SD).

|  | Fig 4B | | | |  |  |  |  |
| --- | --- | --- | --- | --- | --- | --- | --- | --- |
|  | Sham | OA | | On-Low | | On-Mid | On-High |  |
|  | 0.5 | | 6 | 4 | | 4 | 3 |  |
|  | 0 | | 5.5 | 4.5 | | 3.5 | 3.5 |  |
|  | 1 | | 5 | 5 | | 3 | 2.5 | |
|  | 0 | | 5.5 | 5 | | 3.5 | 2 | |
|  | 0 | | 6 | 4.5 | | 3.0 | 1.5 | |
|  | 0.5 | | 5.5 | 4.5 | | 3 | 3 | |
| Mean ± SD | 0.35±0.41 | | 5.55±0.3^***^ | 4.45±0.44^###^ | | 3.45±0.44^###$$^ | 2.35±0.67^###△△^ | |
| F value | 104.7 | | | | | | |  |
| P value | <0.0001 | | | | | | |  |

^***^P < 0.001 vs. Sham group; ^###^P < 0.001 vs. OA group; ^$$^P < 0.01 vs. On-Low group; ^△△^P< 0.01 vs. On-Mid group.

**Table 4.** Ononin reduced the increased levels of IL-1β, TNF-α and IL-6 in OA rat serums (value, Mean ± SD).

| Groups | N | Fig 5 | | |  |
| --- | --- | --- | --- | --- | --- |
|  |  | IL-1β | TNF-α | IL-6 | |
| Sham | 10 | 46.8± 8.72 | 30.8 ± 5.1 | 16 ± 4.49 | |
| OA | 10 | 127.9 ± 10.3^***^ | 96.3 ± 8.8^***^ | 78.3 ± 6.18^***^ | |
| On-Low | 10 | 93.2± 12.04^###^ | 72.5 ± 5.24^###^ | 57.7 ± 3.44^###^ | |
| On-Mid | 10 | 69.7±5.14^###$$$^ | 57.8±4.56^###$$$^ | 42.8±4.31^###$$$^ | |
| On-High | 10 | 51.6±6.33^###△△△^ | 36.5±4.39^###△△△^ | 28±3.9^###△△△^ | |
| F value |  | 141.7 | 189.4 | 260.1 | |
| P value |  | <0.0001 | <0.0001 | <0.0001 | |

***P < 0.001 vs. Sham group; ^###^P < 0.001 vs. OA group; ^$$$^P < 0.001 vs. On-Low group; ^△△△^P < 0.001 vs. On-Mid group. Additional information on data in S3 Table 3-5.

**Table 5.** Quantitative analysis of relative positive cells collagen II (C) and MMP13 (D) in the cartilage samples (value, Mean ± SD).

| Groups | N | Fig 6C、D | |
| --- | --- | --- | --- |
|  |  | Collagen II(%) | MMP-13(%) |
| Sham | 3 | 44.33 ± 5.03 | 3.67 ± 2.08 |
| OA | 3 | 4 ± 1^***^ | 73.67± 5.13^***^ |
| On-Low | 3 | 13 ± 2^#^ | 41.33 ± 3.51^###^ |
| On-Mid | 3 | 20.67±1.53^###$$$^ | 27.67±3.21^###$$^ |
| On-High | 3 | 40.33±2.52^###△^ | 14.33±1.53^###△△^ |
| F value |  | 116.5 | 138.3 |
| P value |  | <0.0001 | <0.0001 |

***P < 0.001 vs. Sham group; ^###^P < 0.001 vs. OA group; ^$^P < 0.001 vs. On-Low group; ^$$^P < 0.001 vs. On-Low group; ^△△^P < 0.001 vs. On-Mid group; ^△△△^P < 0.001 vs. On-Mid group. Additional information on data in S3 Table 6、7.

**Table 6.** Relative protein expressions of collagen II and MMP-13 was qualified by Image-J software. (value, Mean ± SD).

| Groups | N | Fig 6E、F | |
| --- | --- | --- | --- |
|  |  | Collagen II | MMP-13 |
| OA | 4 | 0.18 ± 0.03^***^ | 5.99± 0.2^***^ |
| On-Low | 4 | 0.38 ± 0.05^###^ | 5.15 ± 0.26^##^ |
| On-Mid | 4 | 0.63±0.04^###$$$^ | 3.73±0.34^###$$$^ |
| On-High | 4 | 0.75±0.04^###△△^ | 2.54±0.23^###△△△^ |
| F value |  | 161.4 | 138.3 |
| P value |  | <0.0001 | <0.0001 |

Data are expressed as fold change compared to the Sham group.

***P < 0.001 vs. Sham group; ^##^P < 0.01 vs. OA group; ^###^P < 0.001 vs. OA group; ^$$$^P < 0.001 vs. On-Low group; ^△△^P < 0.01 vs. On-Mid group; ^△△△^P < 0.001 vs. On-Mid group. Additional information on data in S3 Table 8、9.

**Table 7.** Relative phosphorylation levels of ERK, JNK, p38, IκBα, and p65 were qualified by ImageJ software and was normalized by corresponding total protein content. (value, Mean ± SD).

| Groups | N | Fig 7B-F | | | | |
| --- | --- | --- | --- | --- | --- | --- |
|  |  | p-ERK | p-JNK | p-p38 | p-IκBα | p-p65 |
| OA | 4 | 3.08± 0.14^***^ | 2.42 ± 0.1^***^ | 2.6 ± 0.13^***^ | 2.25 ± 0.13^***^ | 4.57 ± 0.36^***^ |
| On-Low | 4 | 2.51 ± 0.2^###^ | 2.02 ± 0.14^###^ | 2.19 ± 0.16^###^ | 1.93 ± 0.08^###^ | 3.28 ± 0.09^###^ |
| On-Mid | 4 | 1.81 ± 0.14^###$$$^ | 1.66 ± 0.09^###$$^ | 1.73 ± 0.12^###$$$^ | 1.63 ± 0.06^###$$^ | 2.27 ± 0.18^###$$$^ |
| On-High | 4 | 1.32±0.1^###△△^ | 1.3±0.03^###△△^ | 1.33±0.04^###△△^ | 1.34±0.05^###△△^ | 1.54±0.1^###△△^ |
| T value |  | 108.4 | 97.32 | 84.66 | 88.77 | 156.4 |
| P value |  | <0.0001 | <0.0001 | <0.0001 | <0.0001 | <0.0001 |

Data are expressed as fold change compared to the Sham group.

***P < 0.001 vs. Sham group; ^###^P < 0.001 vs. OA group; ^$$^P < 0.01 vs. On-Low group; ^$$$^P < 0.001 vs. On-Low group; ^△△^P < 0.01 vs. On-Mid grou. Additional information on data in S3 Table 10-14.
